# Muscle weakness after critical illness: unravelling biological mechanisms and clinical hurdles

**DOI:** 10.1186/s13054-025-05462-z

**Published:** 2025-06-17

**Authors:** Alexandre Pierre, Raphael Favory, Claire Bourel, Michael Howsam, Raphael Romien, Steve Lancel, Sebastien Preau

**Affiliations:** 1 Univ. Lille, Inserm, CHU Lille, Institut Pasteur de Lille, U1167 - RID-AGE - Facteurs de Risque Et Déterminants Moléculaires Des Maladies Liées Au Vieillissement, 59000 Lille, France; 2 Division of Intensive Care, Hôpital Roger Salengro, CHU de Lille, 59000 Lille, France

**Keywords:** ICU survivors, Post-ICU syndrome, Long-term outcome, Muscle weakness, Translational research

## Abstract

Survivors of intensive care unit (ICU) are increasingly numerous because of better hospital care. However, several consequences of an ICU stay, known as post-intensive care syndrome, worsen long-term prognoses. A predominant feature in survivors is reduced muscle strength, mass, and physical function. This leads to lower exercise capacity, long-lasting physical disability, higher mortality risk, and subsequent health costs. While ICU-acquired muscle weakness has been extensively studied these past decades, underlying mechanisms of post-ICU muscle weakness remain poorly understood, and there is still no evidence-based treatment for improving long-term physical outcomes. One hypothesis, among others, could be that the pathophysiology is dynamic over time, differing between the acute ICU and post-ICU recovery periods. This narrative review aims to address the clinical, physiological and biological determinants of persistent muscle dysfunction in ICU survivors, with particular attention to the molecular, cellular and systemic mechanisms involved. Specifically, pre-ICU health factors such as obesity and sarcopenia, ICU-related complications and treatments, and post-ICU management all influence recovery. Dysfunctions in the neuroendocrine, vascular, neurological, and muscle systems contribute as physiological determinants of the muscle weakness. Complex and multifaceted biological mechanisms drive the post-ICU muscle dysfunction with mitochondrial and autophagy dysfunction, epigenetic modifications, cellular senescence, muscle inflammation with altered cell–cell communication, including dysfunction of immune cells, stem cell exhaustion and extracellular matrix remodelling. The review also sheds light on new and innovative therapeutic approaches and discusses future research directions. Emphasis is placed on the potential for multi-approach treatments that integrate nutritional, physical, and biological interventions. Addressing these aspects in a holistic and dynamic manner, from ICU to post-ICU phases, may provide avenues for mitigating the long-term burden of muscle weakness and physical disability in ICU survivors.

**Supplementary Information:**

The online version contains supplementary material available at 10.1186/s13054-025-05462-z.

## Introduction

Despite the increasing age and severity of illness among Intensive Care Unit (ICU) patients, the number of survivors is substantial and rising, reaching approximately 16 million worldwide each year because of better hospital care [1]. However, several long-lasting consequences after ICU stay, known as post-intensive care syndrome (PICS), impair long-term prognoses. A predominant feature in survivors is the reduction of muscle strength during their hospital stay that persists years after ICU discharge and results in reduced exercise capacity, long-lasting physical disability, distress, and higher subsequent mortality risk up to 5 years after discharge [2–4]. It also results in further health costs, as many survivors require rehabilitation and long-term facilities, meaning that PICS is becoming a significant public health challenge [5].

Despite numerous studies describing the long-term ICU consequences on skeletal muscle and physical function, research exploring the physiological and biological mechanisms remains scarce and there is still no evidence-based treatment for improving long-term outcomes. One hypothesis, among others, could be that the pathophysiology is dynamic over time, differing between the acute ICU period and the recovery phase after ICU discharge. In this field, the most significant knowledge is based on experimental models and ICU-surviving patients who experienced sepsis, acute lung injury and mechanical ventilation.

This review provides an integrative and updated perspective on post-ICU muscle dysfunction, going beyond prior work by bridging clinical, physiological, and molecular insights across the ICU and post-ICU continuum. By framing muscle weakness as a dynamic and multifactorial process, the review offers a comprehensive foundation for future therapeutic strategies. The detailed methodology used to conduct this review is described in the supplementary materials (see Additional file 1).

## Long-term muscle and physical consequences

While ICU-acquired weakness (ICUAW), clinically defined by a Medical Research Council (MRC) sum score < 48 in patients with no plausible cause other than critical illness, worsens the short-term consequences (e.g., increased mechanical ventilation duration, length of stay, and in-hospital mortality), it also impacts long-term outcomes [1, 6]. It is well known that after an initial improvement in physical capacities during the first three to six months following ICU discharge, muscle function in survivors reaches a plateau that persists for years thereafter [7]. Long-term studies on ICU survivors indicate that these individuals often suffer from persistent weakness and prolonged physical impairments, with reported prevalence exceeding 60% and 90%, respectively, among sepsis survivors [8]. Within the first year after ICU discharge, survivors exhibit significant declines in muscle maximal and endurance forces [2]. Muscle weakness directly and negatively impacts physical performance and health-related quality of life (QOL) [9]. Physical functioning limitations persist for up to five years, as evidenced by reduced distance in the 6-min walk test and aerobic capacity [4, 10]. Indeed, during the 5-year follow-up of the EPaNIC study, 361 patients underwent cardiopulmonary exercise testing, which revealed a 24% reduction in peak VO_2_ compared with healthy controls. This deterioration in exercise capacity involved muscle limitations in more than 60% of cases [4]. The decline in physical ability can severely impact patient autonomy, with nearly half of those living independently before hospitalization losing this ability six months post-ICU [11]. However, recovery trajectories vary among ICU survivors [12], and while some ICU survivors may experience improvements in functional status, others may never achieve full recovery. Indeed, two-thirds of septic shock survivors had not regained their pre-ICU physical status one year after discharge [10]. The physical recovery is influenced by clinical, physiological, and biological determinants, with long-lasting molecular, cellular, and systemic abnormalities.

## Clinical factors

### Pre-ICU health status

**Fig. 1:**
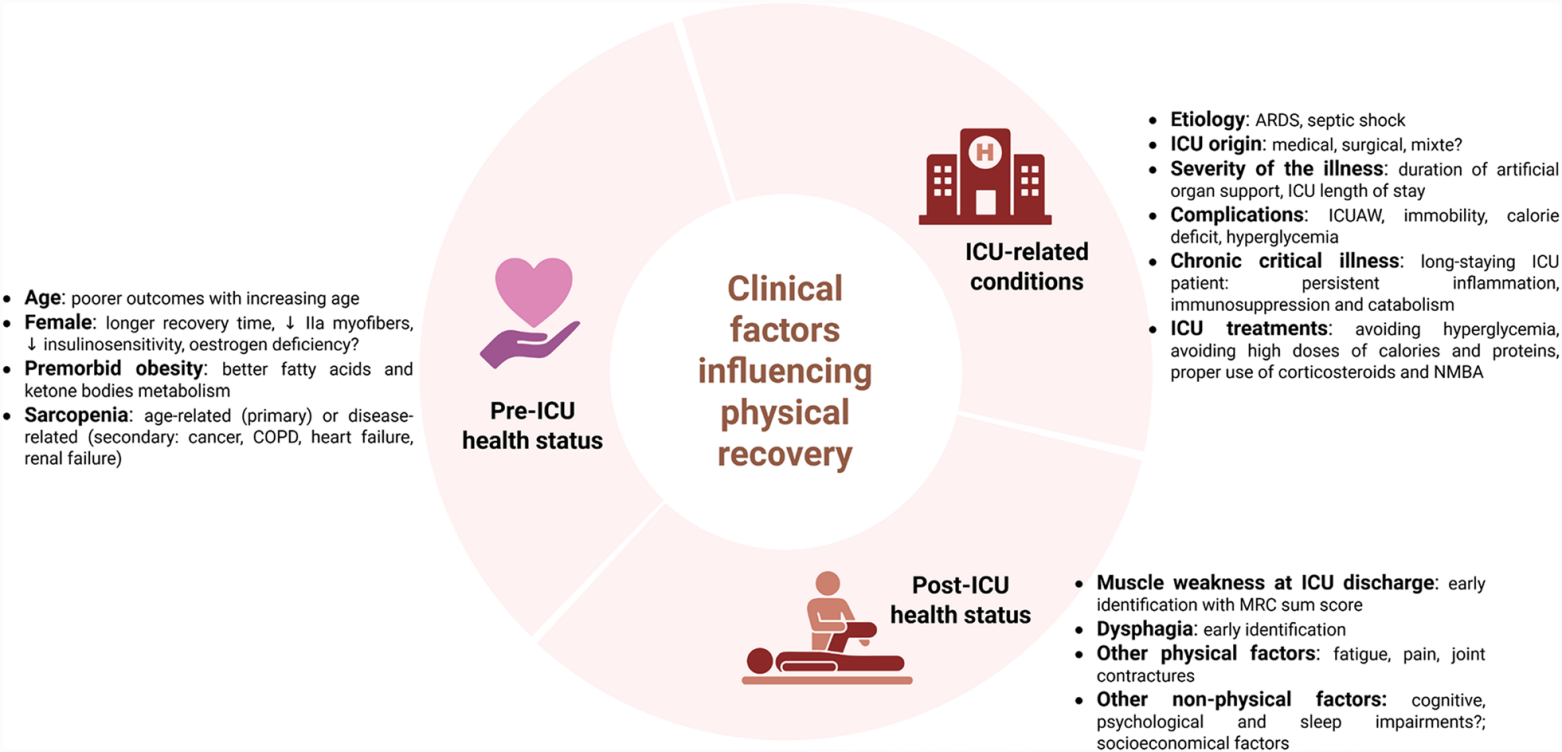
Clinical factors influencing the post-ICU physical recovery. ARDS: acute respiratory distress syndrome, ICU: Intensive Care Unit, ICUAW: ICU acquired weakness, NMBA: neuromuscular blocking agents, QOF: quality of life

Similar to its impact on short-term ICU outcomes, age also affects long-term physical recovery, with older individuals experiencing poorer outcomes [13]. While females are more prone to ICUAW, they exhibit greater impairment of physical function in the long term compared with males [14, 15]. Reduced type IIa myofibers, decreased insulin sensitivity and oestrogen deficiency in critically ill females have been suggested as contributing factors to the observed gender disparities [16–18]. Premorbid obesity is a well-documented factor that attenuates the mortality rate and muscle weakness in both humans and animals [19]. Its effects are independent of the nutritional status (fasting or parenteral feeding) or the activity of the leptin adipokine [20, 21]. It appears to be related to a more efficient mobilization of endogenous fatty acids and an increased availability of ketone bodies, preventing lean tissue wasting [20, 22]. Conversely, pre-existing sarcopenia, defined by the loss of muscle mass, strength, and physical performance due to aging (primary sarcopenia) or underlying diseases (secondary sarcopenia, such as cancer), is a worsening factor for post-ICU muscle weakness, even after adjusting for age and comorbidity levels [23, 24]. This condition better explains the poor long-term functional status than the persistence of muscle mass loss at 3 months after sepsis [23]. Unravelling the causal mechanisms of pre-ICU health status may facilitate the development of targeted strategies (Fig. 1).

### ICU-related conditions

The extent of physical impairment is closely linked to the severity of the illness upon admission and the reason for hospitalization, particularly acute respiratory distress syndrome (ARDS) and septic shock [9, 25, 26] (Fig. 1). The duration of artificial organ support, especially mechanical ventilation, and the length of ICU stay are both associated with poor physical recovery [27, 28]. Importantly, Chronic Critical Illness (CCI), defined by prolonged organ dysfunction lasting more than ten days, has a significant impact on long-term physical function [29]. These long-staying ICU patients frequently suffer from persistent inflammation, immunosuppression, and catabolism syndrome, which further complexifies the pathophysiology [28]. The potential influence of ICU admission origin — surgical *vs.* medical — on post-ICU muscle dysfunction have not been specifically compared. Most available data come from mixed cohorts, which may introduce heterogeneity and limit the interpretation of findings. Future studies are warranted to determine whether distinct processes contribute to muscle dysfunction in surgical versus medical populations (See Additional file 2).

Hyperglycaemia, a well-known risk factor for ICU-acquired weakness (ICUAW) [6], and its treatment with insulin may disrupt neuronal and muscle cell homeostasis by affecting mitochondrial function and autophagy [30]. However, the role of hyperglycaemia in the ICU as either a pathogenic or adaptive response remains debated. Calorie deficit – constituted in the acute phase – is reported to be a risk factor for muscle wasting in ICU survivors, independent of the severity of illness [31]. Nevertheless, early instauration of full feeding does not improve long-term functional outcomes and these patients experience slower physical recovery [32–34]. This may be attributed to anabolism resistance, worsening of hyperglycaemia, insulin needs, and autophagy dysfunction [35, 36]. Conversely, experimental studies demonstrate that the energy deficit does not worsen the muscle phenotype in ICU animals [35, 37–39]. In line with these results, restricting calorie and protein intake (6 kcal/kg/d and 0.2–0.4 g/kg/d versus 25 kcal/kg/d and 1.0–1.3 g/kg/d) in the acute phase (*i.e*. first 7 days) leads to a faster recovery without harmful effects in critically ill patients [40]. Immobility due to prolonged bed rest and inactivity leads to rapid muscle atrophy and exacerbates neuromuscular dysfunction [6]. Studies of the impact of corticosteroids and neuromuscular blocking agents (NMBA) on physical recovery are controversial and may depend on the administration duration, dose, and indication [9, 27, 41, 42]. These data support the proper use of medicines, including minimizing the dose of corticosteroids (Fig. 1).

### Post-ICU health status

Muscle weakness at ICU discharge is a strong predictor of poor physical recovery, underscoring the need to identify high-risk patients early. Assessing the MRC sum score remains crucial before hospital discharge. Survivors with a low MRC score at ICU discharge, particularly those scoring below 55, have a higher risk of long-term mortality and poor outcomes [1, 3, 6]. Importantly, MRC scores at ICU discharge correlate with long-term measures of muscle strength, physical function, and quality of life, even five years after ICU discharge [3]. In addition to peripheral muscle weakness, ICU-acquired dysphagia, negatively influences physical health [43]. Approximately 30% of mechanically ventilated patients develop clinically significant post-extubation dysphagia (PED), which may persist in up to 18% of sepsis survivors three years after ICU discharge [8, 44]. The pathophysiology of PED is multifactorial, encompassing local laryngo-pharyngeal trauma induced by endotracheal intubation, pharyngeal and laryngeal muscles dysfunction, and impaired central regulation of swallowing [45]. PED is associated with delayed resumption of oral feeding, increased risk of malnutrition, delayed physical recovery, prolonged hospital stay, and higher mortality [46, 47]. Expert recommendations now advocate for systematic screening and early management of swallowing disorders in critically ill patients, as part of a comprehensive post-ICU rehabilitation strategy aimed at optimizing nutritional status and functional outcomes [45] (Fig. 1).

**Fig. 2:**
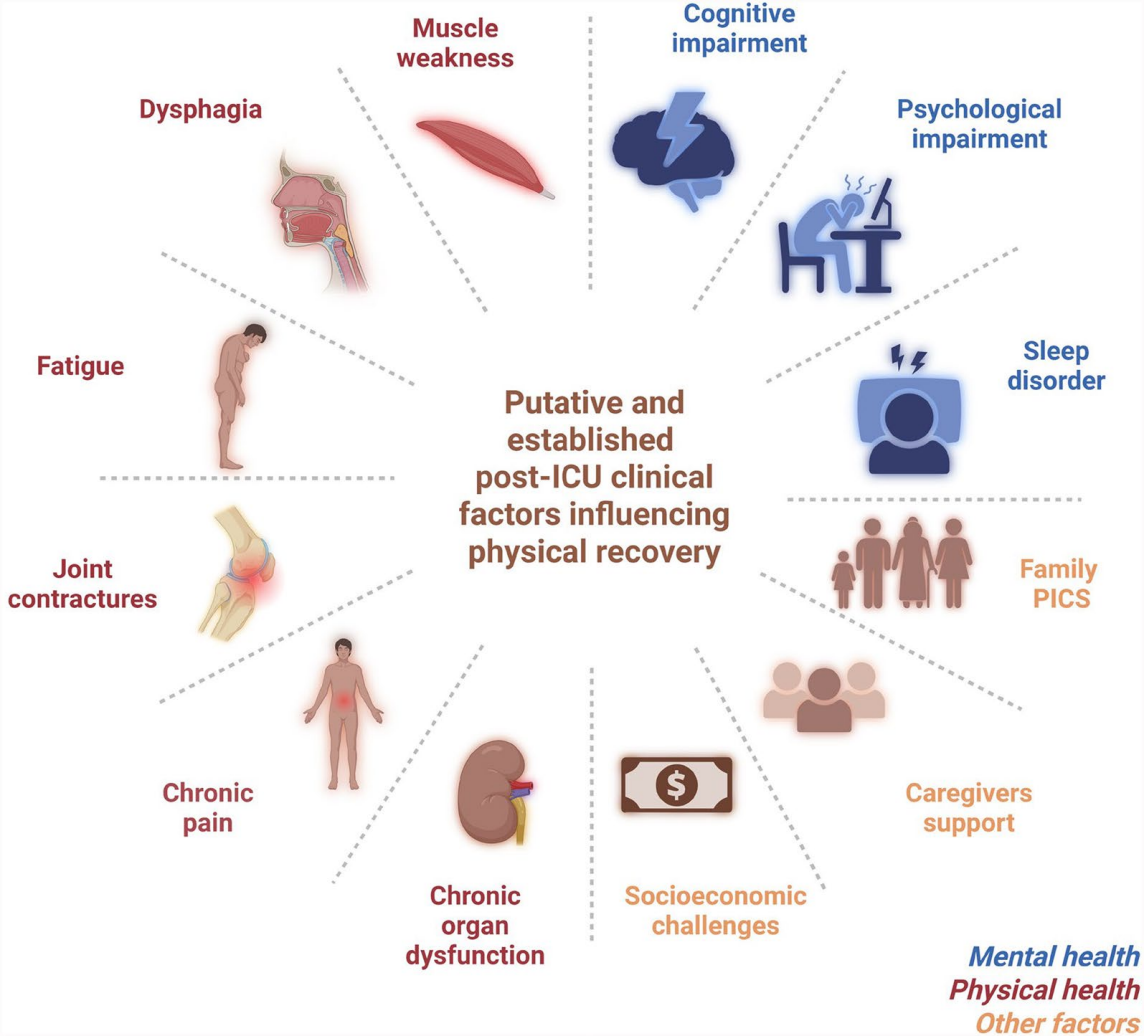
Putative and established post-ICU clinical factors influencing physical recovery. Factors related to physical health are shown in red (left), mental health in blue (upper right), and other factors in yellow (lower right). PICS: post-intensive care syndrome

Beyond muscle pathology, survivors of critical illness face numerous barriers that hinder physical recovery. Fatigue, highly prevalent in ICU survivors may impact physical recovery and is addressed in the “Neurological dysfunction” section. Persistent pain is frequently reported in this population, with up to 66% of ICU survivors develop new chronic pain in the months following discharge [8, 48]. Joint contractures affect over one-third of ICU survivors at hospital discharge and further restrict mobility and complicate rehabilitation [49, 50]. Other new chronic organ dysfunction, such as post-ICU chronic kidney failure, negatively modifies long-term functional outcomes [51, 52]. In addition, poor mental health may adversely influence physical recovery. More than 60% of survivors experience sleep disorders, often persisting for months after discharge [53, 54]. Psychological impairments, including depression, anxiety, and post-traumatic stress disorder, and cognitive impairments, including deficits in attention, memory, and executive function, are observed in more than one-third of ICU survivors at one year [55–58]., Furthermore, ICU survivors face significant socioeconomic challenges, as functional impairments often limit their ability to return to work, leading to substantial lost earnings [59, 60]. Finally, the burden of critical illness extends beyond the patient. Family involvement may be a critical determinant of functional recovery, yet caregivers frequently report limited resources, and a lack of support [61, 62]. Overall, addressing pain control, prevention of contractures, neuropsychological support, family-centered care, and socioeconomic reintegration may improve the long-term physical outcomes in addition to muscle-specific therapies (Fig. 2).

## Physiological determinants

### Neuroendocrine abnormalities

**Fig. 3:**
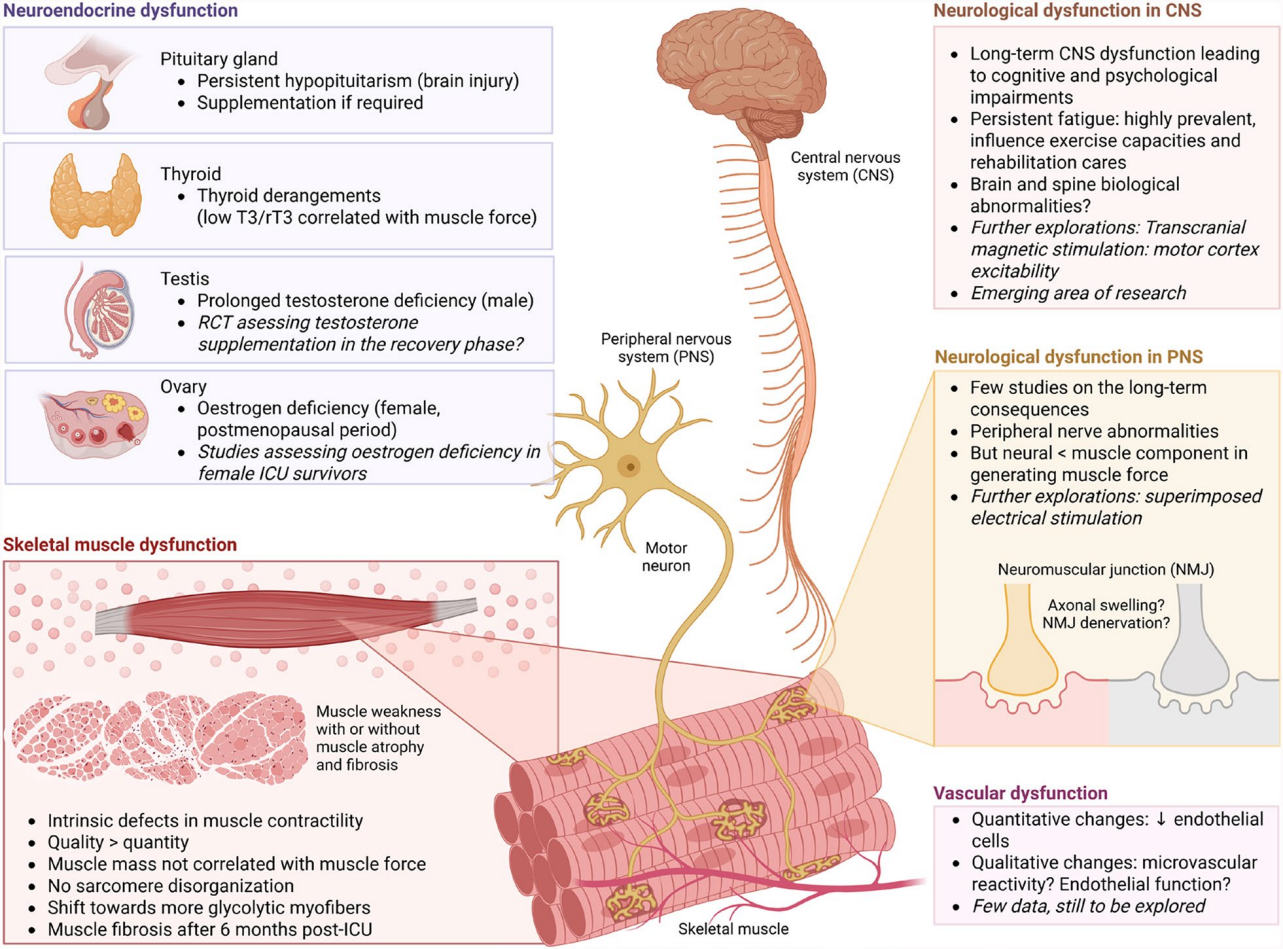
Physiological determinants of the post-ICU muscle weakness at the system level. Further research directions are highlighted in italics. T3: triiodothyronine, rT3: inactive reverse T3, CNS: Central nervous system, PNS: peripheral nervous system, NMJ: neuromuscular junction, RCT: randomized controlled trial

In the early phase (*e.g*. first days), impairment in the peripheral hormone metabolism leads to active secretion from the anterior pituitary, reduced availability of anabolic hormones, and increased availability of catabolic hormones [63]. In the late phase (*e.g.* after a few days), neurohormonal secretions are repressed, primarily due to hypothalamic deficit, with insufficient levels of peripheral hormones such as cortisol [63, 64]. After critical illness, knowledge is scarce. Persistent hypopituitarism lasts for years in patients after traumatic brain injury and is associated with poor physical outcomes and quality of life [65]. In non-brain injured patients, Vanhorebeek et al*.* explored the somatotropic, thyroid, and adrenal axis 5 years after ICU discharge in the EPaNIC follow-up study [66]. While most acute changes resolve, inactive reverse triiodothyronine (rT3) levels and the T3/rT3 ratio remain low, and are correlated with reduced hand grip strength [66]. Whether targeting the long-term thyroid axis dysfunction may improve physical recovery remains to be demonstrated in translational studies (Fig. 3 and Table 1).

**Table 1:** Summary of the dynamic pathophysiology changes from the ICU period to the post-ICU convalescence and further research perspectives

	Current knowledge		Research perspectives	
	Critical illness	Post-critical illness (convalescence)	Better understanding pathophysiology	Therapeutic opportunities
**Physiological determinants**				
Skeletal muscle	• Altered myofiber contractility • Myofiber atrophy • Altered muscle cell excitability • Sarcomere disorganization • Preferential loss of myosin over actin • Preferential loss of Myhc II over Myhc I • Muscle necrosis	• Altered myofiber contractility, independent of myofiber atrophy • Preserved or reduced myofiber size and number • Restored sarcomere organization • Shift towards more glycolytic myofibers (type II) • No muscle necrosis • Muscle fibrosis	Understanding the intrinsic defect in muscle contractility	Optimizing physical therapies during and after critical illness
Peripheral nervous system	Well-documented sensorimotor polyneuropathy	• Neuromuscular junction and axonal abnormalities • But neural < muscle component in force generation	Further physiological assessments of the motor control, such as superimposed electrical stimulation	Optimizing NMES during and after critical illness
Central nervous system	NA	Few data, high prevalence of fatigue, influencing exercise capacities	Further research required to explore the role of the CNS: transcranial magnetic stimulation	NA
Neuroendocrine system	• Repression of neurohormonal secretions • Major thyroid disturbances • Testosterone deficiency • Oestrogen deficiency	• Persistent hypopituitarism (trauma) • Minor thyroid disturbances • Prolonged testosterone deficiency • Prolonged oestrogen deficiency?	Further investigations to elucidate the role of oestrogen in post-critical illness physical recovery	Testosterone supplementation in the early convalescence?
Vascular system	Microvascular dysfunction with reduced reactivity	Few data, reduced number of endothelial cells in preclinical models	Further research required to explore the role of the vascular system	NA
**Biological mechanisms**				
Nuclear and organelle dysfunction				
Anabolism	Anabolic resistance: Reduced muscle protein synthesis Inability to utilize ingested protein for muscle protein synthesis	Feeding responsiveness: Restoration of the protein synthesis pathway governing the muscle mass recovery (Akt-TSC2-mTORC1 signal transduction)	Understanding the dynamics of anabolic resistance to accelerate the muscle mass recovery?	Optimizing nutrition and protein intake according to the anabolic state
Catabolism: ubiquitin proteasome system	• Intense activation of the ubiquitin proteasome system and its proteolysis: • Responsible for acute muscle wasting? • But a balanced activation remains essential for muscle homeostasis?	• Resolution of the ubiquitin proteasome system activation • Resolution of sarcomere organisation	Understanding the dynamics of UPS activation	UPS inhibition probably not effective in improving long-term muscle function
Catabolism: autophagy	• Biological state of autophagy activation: Akt/mTOR inhibition and AMPK activation • Responsible for acute muscle wasting? • But muscle phenotype of autophagy insufficiency • Remains essential for muscle homeostasis	• Biological state of autophagy inhibition: mTOR activation, no AMPK activation • Persistent muscle phenotype of autophagy insufficiency	Evaluating autophagy flux in human ICU survivors	Initiating an autophagy enhancer upon ICU admission?
Mitochondrial function	• Mitochondrial dysfunction • Mitochondrial biomass reduction • Impaired mitochondrial quality control pathways	• Sustained mitochondrial dysfunction • Restored mitochondrial biomass but persistent dysregulation of mitochondrial pathways in humans • Exercise capacity limited by muscle oxygen utilization	• Understanding the dynamics of mtQC • Better characterizing the mitochondrial population in ICU survivors	Initiating mitochondrial enhancer upon ICU admission or in the early convalescence?
Insulin resistance	Impaired insulin signalling leading to defective GLUT4 translocation	Sustained insulin resistance: impaired glucose tolerance and new-onset diabetes	Elucidating molecular mechanisms of post-ICU insulin resistance using preclinical and human models	Metformin in the early convalescence: mitigating insulin resistance via AMPK pathway, influencing other mechanisms (mitochondria, autophagy)
Cellular replication	Not studied	• Cellular senescence: p53-dependent in preclinical models • Participation to muscle low-grade inflammation	Studying replication pathways in humans	Testing senolytics in preclinical models
DNA methylation	Hypomethylation: mitochondrial homeostasis, muscle regeneration, neuromuscular receptors	Persistent and aberrant DNA methylome: physical development (post-ICU paediatric population)	Better characterizing muscle epigenetics in ICU patients and survivors	Modulation of epigenetics in preclinical models
Alteration of cell–cell communication				
• Muscle regeneration	• Muscle damage with necrosis phenotype	• Impaired muscle regeneration and repair • MuSCs dysfunction with mitochondrial alterations in pre-clinical models	• Studying the MuSCs in humans • Understanding the dynamics of intercellular communication and myogenesis program in human ICU survivors using high-resolution temporal and spatial single-cell analysis	• Starting therapeutics before the onset of fibrosis • Antifibrotic drugs in the late convalescence? • Intramuscular injection of mesenchymal stem cells, starting in the early convalescence? • Anti-inflammatory drugs: probably ineffective within ICU, worth considering in early convalescence?
Muslce repair	NA	• Failure to regulate the myogenesis program in survivors with sustained muscle atrophy: excess proliferation and differentiation of myoblasts • Failure of cell–cell coordination leading to excessive extracellular matrix deposition leading to muscle fibrosis muscle		
Muscle inflammation	Severe muscle inflammation: immune cells infiltration and high level of cytokines (IL6, IL1β, TNFα) and DAMPs	• Sustained low-grade inflammation: immune cells infiltration and moderate level of cytokines and DAMPs • Profund reconfiguration of immune cells interaction within the muscle microenvironment		

Early convalescence corresponds to the period immediately following ICU discharge, while late convalescence begins six months after ICU discharge

Critically ill male patients often experience significant and prolonged testosterone deficiency, which occurs rapidly upon ICU admission and lasts after ICU discharge [67]. Hypogonadism has been associated with low muscle mass and function in ICU patients [17]. Testosterone or analog treatments alleviate muscle catabolism in severely burned patients [68] and improve muscle phenotype in septic animals [69]. Whether testosterone may overcome the anabolic resistance remains to be demonstrated in non-burn ICU survivors. As testosterone or analog treatment appears to be safe [70], RCTs assessing its effect in the recovery phase are warranted.

Female sex is a well-established risk factor for ICUAW and poor physical recovery [14, 15], potentially linked to oestrogen deficiency. Postmenopausal women experience a chronic decline in oestrogen levels, which is associated with accelerated muscle mass and strength loss compared to men [71]. Hormone replacement therapy has been shown to preserve muscle function in this population [18]. Oestrogen deficiency impairs satellite cell activation, promotes muscle fibre atrophy, and delays regeneration after injury [72]. While younger women may benefit from endogenous oestrogen, critical illness itself may transiently suppress gonadal function [17], potentially reducing this protection. Thus, oestrogen deficiency — especially in older female ICU survivors — may represent a relevant but underrecognized factor contributing to long-term muscle weakness [73]. Further investigations are warranted to elucidate the role of oestrogen in post-critical illness physical recovery (Fig. 3 and Table 1).

### Vascular dysfunction

Endothelial dysfunction plays a role in the pathogenesis of ICUAW. However, studies have focused on the first hours of critical illness, and little is known about the convalescent phase [74]. Although microvascular dysfunction improved throughout ICU stay [75], microvascular reactivity defects remain at ICU discharge [76]. In sepsis-surviving mice, quantitative changes are observed with a reduced number of endothelial cells [77]. Given the existing endothelial-myocyte crosstalk, further research is required to explore the role of the vascular system in long-term physical recovery (Fig. 3).

### Neurological dysfunction

Peripheral nerve injuries may play a role in the pathophysiology of long-lasting muscle weakness, as neuromuscular junction and axonal abnormalities are observed in animal models of prolonged ICU stay [78]. Nevertheless, in situ contractility experiments with both direct muscle stimulation and indirect nerve stimulation indicate that the neural component plays a minimal role in reduced muscle force. Instead, alterations in the muscle component appear as the primary mechanism [78]. In humans, electromyography (EMG) and nerve conduction studies indicate electrophysiological evidence of myopathy in 79% of ICU survivors, with those affected experiencing more severe weakness [79]. Physiological assessment, combining surface EMG and ergometry, suggests that the reduced contractility in human ICU survivors one year after hospitalization originates in muscle tissue rather than the nervous system [2]. Further physiological assessments of the motor control, such as superimposed electrical stimulation and transcranial stimulation, may help in deciphering other neurological mechanisms underlying the muscle weakness (Fig. 3 and Table 1).

Even if muscles and nerves are functionally and structurally intact, the decreased muscle activation by central nervous system (CNS) can lead to muscle weakness and fatigue. Fatigue is defined as “an overwhelming sense of tiredness, lack of energy and feeling of exhaustion, fatigue relates to a difficulty in performing voluntary tasks” [80]. Fatigue is commonly reported by ICU survivors, in more than 57% up to 5 years after discharge, with women more likely to be affected than men [81–84]. Fatigue is a determinant of physical performance, limits exercise capacity and influences rehabilitation care [85]. While fatigue is prevalent after critical illness, the underlying mechanisms have not been studied. Post-infectious myalgic encephalomyelitis/chronic fatigue syndrome (ME/CFS) is a complex and debilitating disorder following an infection, characterized by fatigue that cannot be explained by any underlying medical condition. Wallit et al*.* recently demonstrated that patients with ME/CFS exhibited dysfunction in integrative brain regions, decreased parasympathetic activity, and dysregulation of catecholamine pathways, which correlate with reduced muscle strength [86]. While these findings in ME/CFS patients cannot be directly applied to ICU survivors, these data suggest that CNS determinants may play a role in post-sepsis fatigue. In conclusion, much research remains to be done in this field to address significant knowledge gaps that require further investigation [87] (Fig. 3 and Table 1).

### Muscle dysfunction

The muscle component of ICUAW is characterized by two key features: (1) altered myofiber contractility, and; (2) myofiber atrophy (reduced myofiber size and number), with the following hallmarks: altered muscle cell excitability, sarcomere disorganization, preferential loss of myosin over actin, and greater loss of MyHc II (fast-twitch fibers) than Myhc I (slow-twitch fibers) – features not observed in simple bedrest conditions [39, 88, 89]. After critical illness, myofiber contractility and atrophy can be dissociated. Indeed, both animal and human studies have shown persistent muscle weakness despite muscle mass restoration in ICU survivors [84, 90, 91]. In addition, Dos Santos et al*.* found that muscle mass did not correlate with muscle force and even the weakest individuals exhibited restored muscle mass [79]. While sarcomere disorganization and myofiber cross-sectional area is restored in ICU survivors, a shift toward more glycolytic myofibers has been observed [84]. Normalizing muscle force to myofiber cross-sectional area did not alter results, indicating intrinsic contractile dysfunction in ICU survivors, independent of muscle mass. Based on these observations, the biomechanical characteristics of muscle fibres may be at least as important as their quantity (Fig. 3). Contributing mechanisms may include abnormalities in calcium handling, sarcolemma excitability, mitochondrial dysfunction, metabolic imbalances, post-translational modifications in contractile proteins, and fibrosis.

## Biological mechanisms

### Nuclear and organelle dysfunction

#### Insulin resistance and impaired glucose metabolism

Insulin resistance is a hallmark of critical illness, triggered by systemic inflammation and stress-induced hormonal changes. Hyperinsulinemic-euglycemic clamp studies have shown up to a 70% reduction in insulin sensitivity in ICU patients, closely associated with illness severity [92]. Impaired insulin signalling leads to defective GLUT4 translocation to the sarcolemma, thereby limiting glucose utilization in skeletal muscle [93]. Insulin resistance may not fully resolve after ICU discharge. ICU survivors often exhibit persistent glucose metabolism alterations, including impaired tolerance and new-onset diabetes (up to 17% at five years, with elevated risk up to five fold) [94–96]. Several studies have highlighted the association between insulin sensitivity and muscle outcomes in ICU patients. Weber-Carstens et al. observed that in patients with ICUAW, skeletal muscle exhibited resistance to insulin stimulation, which was associated with blunted muscle glucose uptake and more profound muscle fibre atrophy [93]. Insulin sensitivity in ICU patients correlates positively with muscle strength at awakening and at ICU discharge [97], underscoring the link between metabolic and functional recovery. While muscle activation strategies during the ICU stay, such as protocolized physiotherapy and electrical muscle activation, have shown limited effects on insulin sensitivity [97], the post-ICU period may offer a more favourable window for intervention. As anabolic resistance subside, targeted exercise programs may enhance insulin sensitivity and support functional recovery. Similarly, initiating metformin during early convalescence could help reduce post-ICU insulin resistance and contribute to mitigating persistent metabolic disturbances in the skeletal muscle of ICU survivors [98]. Further studies are needed to explore the metabolic benefits of rehabilitation strategies during the post-critical illness period (Table 1).

#### Mitochondrial dysfunction

**Fig. 4:**
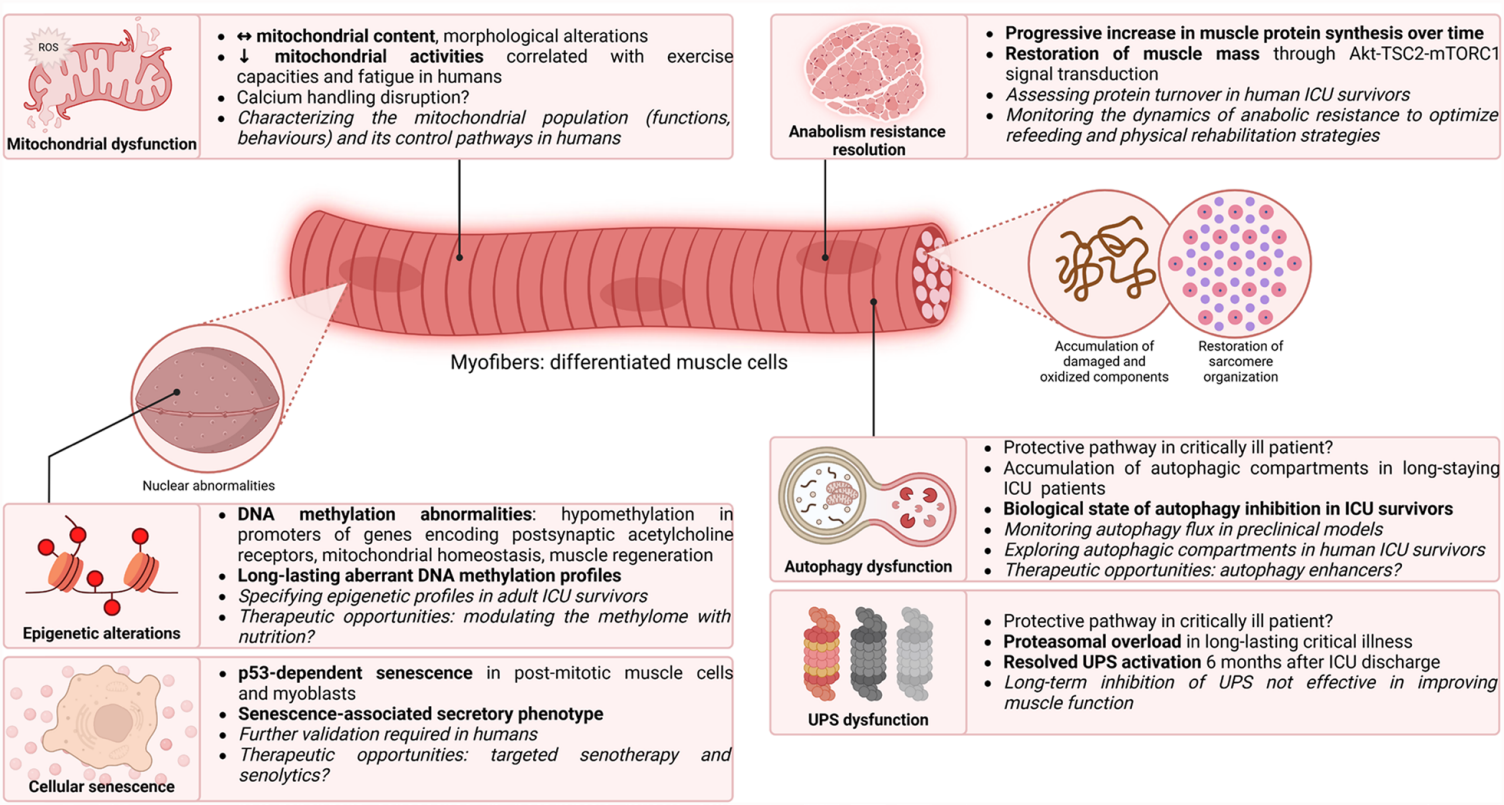
Biological mechanisms underlying post-ICU muscle weakness at the cellular and molecular levels: nuclear and organelle dysfunction. Further research directions are highlighted in italics. ICU: intensive care unit, UPS: ubiquitin proteasome system

Firstly described in human skeletal muscle during septic shock by Gasparetto et al*.* in 1983 [99], mitochondrial dysfunction has been particularly studied in critically ill patients since the early 2000s [100]. In 2002, Brealey et al*.* demonstrated that skeletal muscle energy failure was associated with a poor prognosis and that complex I activity was inversely correlated with the severity of septic shock [101]. The mitochondrial population of skeletal muscle is characterized by reduced mitochondrial biomass and impaired mitochondrial quality control pathway in critical illness [102]. In 2016, Dos Santos et al*.* investigated this hypothesis, among others, and reported no difference in quantitative mitochondrial content, observed by electron microscopy, in ICU survivors 6 months after discharge compared with age- and sex-matched healthy volunteers. However, the authors did not study mitochondrial ultrastructure or morphology [79]. In these same patients, transcriptomic analysis revealed that mitochondrial-related pathways were dysregulated in the post-ICU period [103]. Mitochondrial-related master micro-RNA (miRNA) regulators influence the transcriptomic response and correlate with muscle outcomes [104]. In 2019, using a murine model of cecal slurry injection, Owen et al*.* described alterations in mitochondrial ultrastructure, respiration and enzymatic activities up to 1 month after sepsis, which paralleled muscle oxidation and nitrosylation [90]. Up to five years after ICU discharge, the aerobic capacity of patients was reduced mainly due to muscle limitation [4]. Mart et al*.* showed that human ICU survivors exhibited exercise responses similar to those observed in non-critically ill patients with mitochondrial myopathies. Oxygen utilization measurements were strongly correlated with VO_2_ peak values and the 6-min walk test, suggesting that the exercise capacity may be limited by oxygen utilization [105]. Recently, Mayer et al*.* demonstrated mitochondrial alterations in eleven humans 9 months after ICU discharge. Survivors exhibited reduced mitochondrial complex II activity that correlated with decreased exercise capacity and increased fatigue [84]. Finally, no published study to date has directly demonstrated alterations to the function and behaviour of mitochondria in human ICU survivors [106, 107]. Characterizing the mitochondrial population and its quality control pathways using muscle biopsy is warranted in ICU survivors to achieve a comprehensive understanding of the cellular and molecular processes involved. This could further facilitate the development of therapeutic approaches to counteract muscle weakness (Fig. 4 and Table 1).

#### Cellular senescence

Senescence, is defined as a stable cell-cycle arrest in response to cellular stress, with phenotypic changes such as mitochondrial dysfunction. It can become pathogenic in certain conditions, particularly in survivors of critical care [108, 109]. It leads to the secretion of pro-inflammatory, pro-apoptotic, and pro-fibrotic molecules, known as the senescence-associated secretory phenotype (SASP), perpetuating accelerated aging through low-grade inflammation [110]. While cellular replication has not been studied in the acute phase of critical illness, Chen et al*.* demonstrated that muscle p53-dependent senescence occurred in sepsis-surviving animals. Importantly, metformin attenuated muscle cellular senescence and loss of muscle strength [107]. Metformin is a well-documented senotherapy acting through mechanisms including AMPK activation, mTOR inhibition, autophagy enhancement, and promotion of mitochondrial biogenesis, resulting in anti-oxidative and anti-inflammatory effects [98]. However, metformin's broad activity in the human body and challenges in its use in critically ill patients suggest targeted therapy may be preferable. In this regard, targeted senolytics — drugs that eliminate senescent cells — were first used in humans in 2019 to improve physical performance [111]. While this research field is rapidly expanding, it holds promise for ICU survivors [108]. Future studies should focus on assessing senescence signalling pathways in the skeletal muscle of ICU survivors to validate the relevance of this hypothesis (Fig. 4 and Table 1).

#### DNA methylation abnormalities

Epigenetic abnormalities, by disrupting gene expression and cellular phenotype, are linked to human diseases and affect critically ill patients through modifications of the entire epigenetic network [112]. Van Dyck et al*.* conducted the largest study on the subject and studied the skeletal muscle DNA methylation of 172 critically ill patients in 2022. They identified two hypomethylated regions in the promoters of the HIC1 and NADK2 genes in skeletal muscle, which are crucial for muscle regeneration, postsynaptic acetylcholine receptors, and mitochondrial homeostasis, respectively [113]. While the muscle methylation status was assessed during the ICU stay (day 8 ± 1), no data are available in ICU survivors. Modulation of aberrant DNA methylation and the subsequent transcriptional program may be an innovative approach to alleviate the global, long-term epigenetic issues in ICU survivors (Fig. 4 and Table 1).

#### Anabolism resistance

Interventions aimed at promoting anabolism to counteract catabolism and restore muscle mass have been examined since 1999. Notably, the first large, double-blind RCT assessing growth hormone in patients with prolonged critical illness reported a two-fold increase in mortality compared with placebo [114], suggesting anabolism resistance. This issue remains relevant two decades later, as Chapple et al*.* have recently demonstrated the inability of critically ill patients under mechanical ventilation to utilize ingested protein for muscle protein synthesis [115]. Anabolism resistance is now a well-documented phenomenon in critically ill patients, yet it remains unclear whether this condition persists in survivors. In long-staying ICU patients, muscle protein turnover is negative between days 10 and 20 after admission, but the synthesis rate subsequently increases to reach the breakdown rate, achieving an equilibrium in protein balance by days 30 to 40 [116]. Crowell et al. identified the Akt-TSC2-mTORC1 pathway as a key driver of muscle mass recovery in post-septic mice, independently of AMPK activity. Muscle protein synthesis, initially suppressed, doubled during recovery, coinciding with body weight restoration [91, 117]. These data demonstrate that the anabolic resistance progressively fades during the period of recovery. Nevertheless, studies assessing protein turnover in human ICU survivors are currently lacking. Understanding the dynamics of anabolic resistance in humans is essential for clinicians, as the transition from this state toward responsiveness may drive the appropriate refeeding and physical rehabilitation (Fig. 4 and Table 1).

#### Proteasome dysfunction

The ubiquitin–proteasome system (UPS), intensely activated in critically ill patients, is directly responsible for acute muscle wasting [118]. Experimentally, atrophy-related genes, including E3 ubiquitin ligases, were highly up-regulated in sepsis mice, partly explained by reduced calorie intake [37]. Long-term critical illness led to a necrotizing muscle phenotype linked to proteasomal overload, indicating a build-up of inadequately degraded proteins by the UPS [119]. Additionally, in an experimental extensive-burn model, both early and late pharmacological inhibition of the proteasome with bortezomib reduced the hypermetabolic muscle response but resulted in increased mortality [120]. Although proteolysis seems harmful to muscle mass, its balanced activation remains essential for muscle homeostasis [121]. Finally, while UPS activation and sarcomere destruction were observed in all ICU survivors 7 days post-discharge, these conditions were fully resolved 6 months later [79]. Overall, inhibition of the UPS does not seem to be an effective therapeutic strategy for improving the long-term physical capacity (Fig. 4 and Table 1).

#### Autophagy dysfunction

Damaged cellular components accumulate during critical illnesses due to inflammation and oxidative stress in the skeletal muscle [1]. Autophagy, a highly evolutionarily conserved process, recycles unnecessary or dysfunctional components through a lysosome-dependent degradation [122]. Nonetheless, the specific role of autophagy in ICU patients remains poorly understood, especially in skeletal muscle [123]. In the acute phase, muscle autophagy is mainly activated through Akt/mTOR inhibition and AMPK activation [123] and may reduce muscle mass. Thus, inhibiting catabolic pathways could prevent muscle wasting in the short term, in preclinical models [124, 125]. However, this does not mean that the overall effect is beneficial on muscle function in the long term. Long-stay ICU patients accumulated up to tenfold more immature or unfused autophagosomes than matched controls, suggesting an insufficient activation of autophagy [126]. Pharmacological or genetic inhibition of autophagy worsened the course of sepsis with increased mortality rate and disease severity [127–129]. In the recovery period, the muscle-specific and inducible deletion of Atg7 worsens the muscle outcomes in sepsis-surviving mice [129]. Transcriptomic analyses revealed that it strongly activates other catabolism pathways, including the UPS, leading to a more severe atrophy. Crowell et al*.* reported a biological state compatible with autophagy inhibition in sepsis survivors [117]. In line with these results, rapamycin, an autophagy enhancer, improved muscle contractility in sepsis-surviving mice 7 days after CLP and resuscitation [128]. Human ICU survivors exhibited a sustained increase in Beclin-1, an autophagy-related protein involved in the initiation of the process, both 7 days and 6 months after intensive care [79]. However, these studies were not designed to specifically evaluate the mechanisms of autophagy. Observations of muscle autophagic compartments have yet to be performed in human ICU survivors. Taken together, maintaining autophagy homeostasis may be indispensable to promote long-term muscle health in ICU survivors (Fig. 4 and Table 1).

### Altered cell–cell communication

#### Muscle inflammation and immune cell dysfunction

Sustained systemic inflammation after critical illness is well-documented and has been associated with poor physical outcomes in human ICU survivors [130]. Muscle cells contribute to the inflammatory response in experimental sepsis, as specific muscle and inducible Il6 deletion regulates systemic immune cell trafficking and cytokines [131]. Muscle is prone to inflammation in ICU survivors. When infection had completely resolved, skeletal muscle in survivors exhibited an increase in Il1b, Il18, Tnfα, Il6, Il10 transcripts to moderate levels compared with controls [117]. Muscle transcriptomic analysis revealed that pathways related to inflammatory responses were associated with reduced muscle strength at both 7 days and 6 months after ICU discharge [103]. Quantitative Magnetic Resonance Imaging identified persistent myostructural abnormalities in human ICU survivors, reflecting muscle inflammation and fatty infiltration, both correlated with muscle weakness [132].

**Fig. 5:**
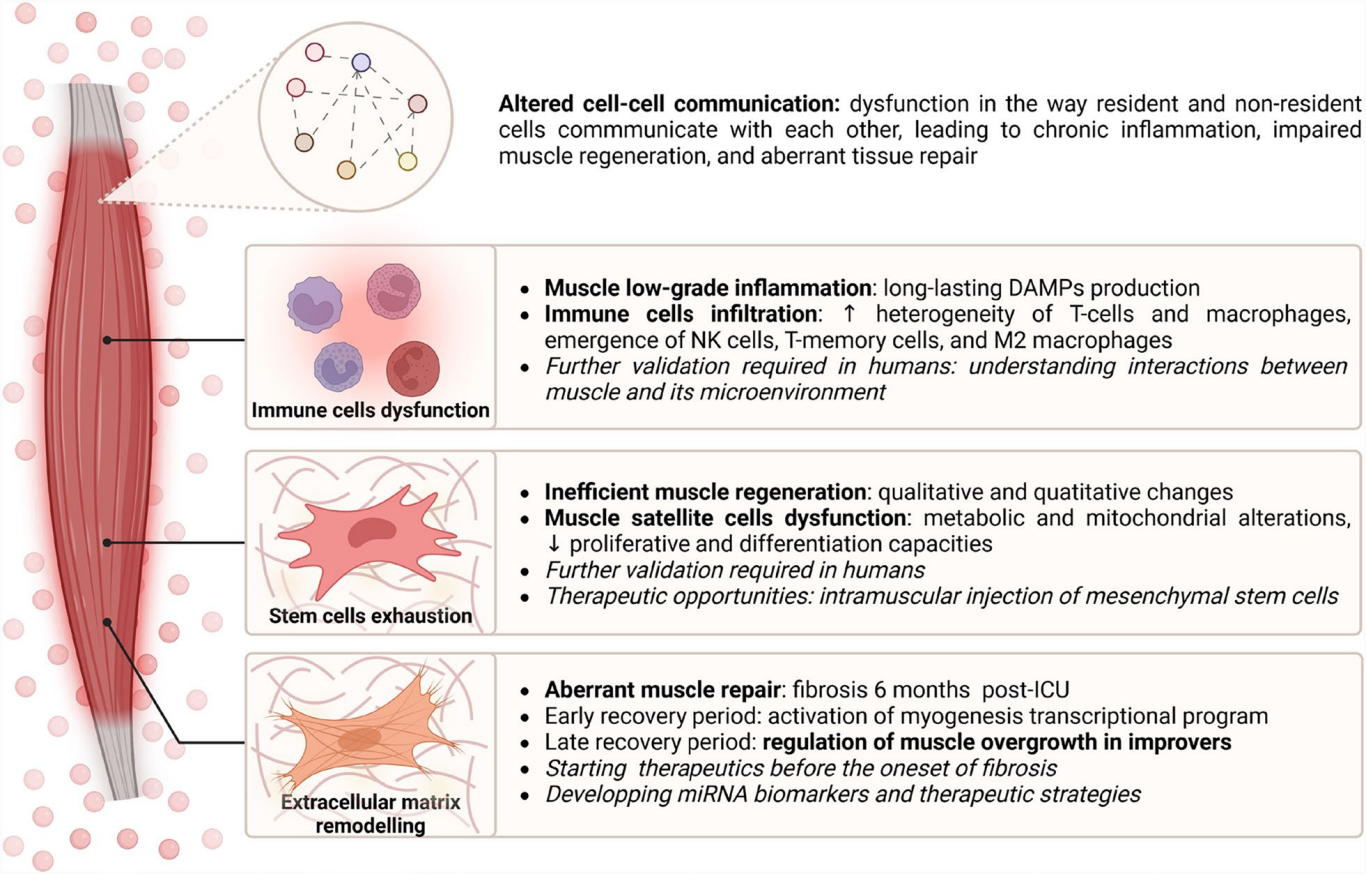
Biological mechanisms underlying post-ICU muscle weakness at the cellular and molecular levels: alteration of intercellular communication. Further research directions are highlighted in italics. DAMPs: damage-associated molecular patterns, NK cells: natural killer cells, miRNA: micro-RNA

Muscle inflammation can also be related to immune cell infiltration. Nakanishi et al*.* demonstrated that infiltration of neutrophils in muscles led to muscle weakness in sepsis-surviving mice [133]. Dos Santos et al*.* found macrophage infiltration in human ICU survivors 7 days after discharge, but which were no longer present at 6 months [79]. Nevertheless, macrophage infiltration has been shown to persist for up to 9 months in COVID-19 ICU survivors [84]. The communication between cells is also of primary importance in maintaining the homeostasis and function of tissue. Interactions between mononuclear cells and signals from immune and mesenchymal interstitial cells are crucial for muscle repair. Disruptions in this network can lead to chronic muscle disorders, resulting in muscle inflammation, fibrosis, adipose infiltration, and muscle atrophy [134]. However, little is known on the subject in the field of critically ill patients. To date, only one single-cell RNA sequencing study has been conducted on the skeletal muscle of surviving mice one month after sepsis, and none have been published to our knowledge on human ICU survivors. The cellular composition of the muscle microenvironment elicits a unique signature with new cell populations. Quantitative analyses of muscle cell populations reveal reduced endothelial cells, fibroblasts, and myogenic cells, alongside increased dendritic cells, neutrophils, and T-cell/macrophage heterogeneity, with emergence of NK cells, T-memory cells, and M2 macrophages [77]. Cell–cell communication was significantly altered, with enhanced signaling toward neutrophils and reduced toward fibroblasts [135]. Inflammation-related pathways were highly dysregulated, notably with strong upregulation of DAMP-associated genes in multiple cell types. Collectively, these data implicate fibroblasts, endothelial cells, neutrophils, T-cells, and DAMP-related signaling in skeletal muscle microenvironment remodeling post-sepsis, likely reflecting altered intercellular communication that may promote chronic inflammation and impaired repair. These findings require validation in humans, particularly regarding immune cell landscape and intercellular communication in skeletal muscle of ICU survivors. Understanding the interactions between muscle cells and their microenvironment seems crucial for elucidating the mechanisms underlying sustained low-grade inflammation and its impact on physical functioning [136] (Fig. 5 and Table 1).

#### Aberrant muscle repair and extracellular matrix remodelling

Following acute tissue injury, inflammatory cells, and stem cells coordinate to restore tissue homeostasis depending on local and systemic signals [137]. However, it fails in some conditions, leading to excessive extracellular matrix deposition and ultimately fibrosis, which impacts muscle contraction. Muscle fibrosis has been described in sepsis-surviving mice [107] and human ICU survivors 6 months after discharge [103]. Walsh et al*.* identified a network of genes involved in the extracellular matrix remodelling that was inversely correlated with muscle strength, and distinct miRNA signatures between 7 days and 6 months post-ICU discharge [103, 104]. Interestingly, Walsh et al*.* identified different miRNA profiles between ICU survivors with recovered muscle mass (improvers) and those with persistent muscle atrophy at 6 months (non-improvers). Improvers exhibited increased expression of two key miRNA regulators known to negatively regulate myoblast activation, compared with non-improvers, suggesting that these miRNAs may play a role in preventing excessive muscle growth. Overall, Walsh et al*.* provide strong data that aberrant muscle repair and extracellular matrix remodelling influence muscle weakness after critical illness. A better understanding of the miRNome of ICU survivors could provide the basis for developing miRNA therapeutic strategies in the future. Since patients reached a muscle function plateau at 6 months post-ICU discharge [7], these results also underline that interventions aiming to improve muscle weakness should likely start in the early convalescence phase, before the onset of fibrosis (Fig. 5 and Table 1).

#### Impaired regenerative capacities

Maintaining regenerative capacity after an injury is fundamental as it enables the restoration of muscle homeostasis through the activation, proliferation, and differentiation of muscle satellite cells (MuSCs). Human ICU survivors exhibited reduced satellite cell content and dysregulated muscle regeneration pathways 6 months after ICU discharge [79, 103]. Sepsis-surviving mice showed persistent necrosis and fibrosis after muscle injury, linked to MuSC metabolic and mitochondrial dysfunction impairing regeneration [138]. Post-sepsis MuSCs exhibited reduced proliferation and differentiation capacities in vitro. Additionally, RNA-seq analysis revealed a mitochondrial defect signature in MuSCs 28 days post-sepsis [139]. In conclusion, both quantitative and qualitative changes alter MuSCs homeostasis in the recovery phase, leading to impaired muscle regeneration. Mesenchymal stem cells (MSCs) of the skeletal muscle are multipotent stromal cells residing in muscle tissue that can differentiate into various cell types, playing key roles in driving muscle repair [140]. Rocheteau et al*.* successfully treated sepsis-surviving mice with intramuscular injection of MSCs [138], restoring muscle mass and function by improving the muscle regeneration capacities. Thus, MSCs grafting appears to be a promising therapy for ICU patients suffering from physical disabilities. However, the lack of data on the functionality of human MuSCs in the post-ICU period currently limits the feasibility of conducting randomized controlled clinical trials for this therapy (Fig. 5 and Table 1).

## Management of post-ICU muscle weakness

### Preventive approaches

Sarcopenia represents the main modifiable component of pre-ICU health status, and addressing this condition may contribute to improving long-term physical outcomes among ICU survivors. Several modifiable risk factors, including low physical activity levels, sedentary behaviour, and inadequate nutritional intake, have been identified as key contributors to sarcopenia and should be systematically screened for [24, 141]. Once sarcopenia is confirmed, targeted interventions combining resistance exercise, nutritional optimization (particularly sufficient protein intake of 1 to 1.5 g/kg/day), and reduction of sedentary behaviour have demonstrated efficacy in preventing or mitigating adverse health outcomes [24, 141, 142] (Table 2).

**Table 2:** Overview of current evidence on clinical risk factors and management strategies for muscle weakness across different phases of critical illness (pre-ICU, ICU stay, and post-ICU), along with identified gaps and directions for future research

	Main clinical factors	Current preventive and therapeutic approaches	Further research perspectives
Pre-existing conditions	• Age • Female • Premorbid obesity ( +) • Sarcopenia: primary (aging) and secondary (COPD, heart failure, renal failure, diabetes, others)	• Identification of sarcopenia risk factors: low physical activity, sedentary behavior, inadequate nutritional intake • Early detection of sarcopenia (SARC-F questionnaire) and formal diagnosis of sarcopenia • Sarcopenia-related interventions: sufficient protein intake 1 to 1.5 kcal/kg/day, resistance exercise, public health policies	• Better understanding the causal mechanisms of pre-ICU health status may facilitate the development of targeted strategies
ICU conditions	• ARDS, septic shock • Severity of illness upon admission • Duration of artificial organ support, especially mechanical ventilation, and ICU length of stay • Chronic critical illness (prolonged organ dysfunction > 10d) • Hyperglycemia, calorie deficit, immobility	*General management* • ABCDEF bundle: reducing ICU length of stay • Proper use of corticosteroids and NMBA • Diagnosis of ICUAW Physical therapy • Protocolized, early (within 72h), adapted (according to patient’s resilience and hemodynamic status) and progressive mobilization *Nutrition* • Avoiding hyperglycemia (insulin if > 1.8 g/dl) • Avoiding high doses of calories and proteins	*Physical therapy* • Determining the optimal dose (duration, intensity, frequency) of early mobilization? • Understanding why NMES improves muscle mass but not muscle strength and why FES doesn’t improve muscle health *Nutrition* • Hypocaloric nutrition in the early phase • Non-carbohydrate-based nutrition: ketogenic enteral nutrition?
Post-ICU conditions	• Being weak at ICU discharge (MRC < 55) • Swallowing disorders at ICU discharge • Non-muscle physical factors: new chronic pain, sustained fatigue, joint contractures • Non-physical factors: cognitive, psychological, sleep disorders? • Family and caregivers consequences? • Socio-economic challenges?	*General management* • Identification of weak survivors at ICU discharge (MRC sum score) and post-extubation dysphagia • Diagnosis of post-ICU muscle weakness *Physical therapy* • Limited evidence for any intervention *Nutrition* • Limited evidence for any intervention • If swallowing disorders: dietary texture modification, compensatory maneuvers	*General management* • Holistic approach: addressing pain control, prevention of contractures, neuropsychological support, family-centered care, and socioeconomic reintegration *Physical therapy* • RCT assessing structured exercise training: increasing cumulative exercise dose from ICU period to post-ICU convalescence, including ward hospitalization and home convalescence • Determining the optimal exercise type (resistance, aerobic, balance, others) and dose (duration/volume, intensity, frequency) • Muscle activation strategies in the post-ICU period may be reconsider given the dynamic pathophysiology *Nutrition* • Protocol-guided individualized dietitian-based nutrition • If swallowing disorders: consider pharyngeal electrical stimulation (using a gastric feeding tube)

### Curative approaches

#### General management

Implementing evidence-based approaches to minimize the duration of ICU stay appears essential in preventing long-term physical disabilities in ICU survivors. One of the most widely recommended strategies is the ABCDEF bundle, a multimodal, patient-centred approach designed to reduce ICU-acquired complications. The bundle includes: A — Assess, prevent, and manage pain; B — Both spontaneous awakening and breathing trials; C — Choice of analgesia and sedation; D — Delirium assessment and management; E — Early mobility and exercise, and; F — Family engagement and empowerment. Adherence to the ABCDEF bundle is linked to shorter ICU stay, less delirium, reduced ventilation duration, and better recovery, with a dose–response relationship indicating greater benefits with higher compliance [143–145] (Table 2).

#### Physical therapy

Although widely studied in critically ill patients, physical therapy shows limited evidence of long-term functional benefit. (1) Despite heterogeneity in results, the overall effect of studies assessing protocolized early and progressive mobilization within the ICU is favourable regarding short-term physical outcomes (e.g. muscle strength at ICU discharge, mechanical ventilation duration and length of stay). While the benefits of early active mobilization are transient and appear not to affect long-term outcomes (e.g. physical recovery or quality of life) [146–156], current guidelines strongly recommend starting the early mobilization, “adapted to the patient’s resilience and general condition “, within the first days in the ICU [157]. (2) Evoked muscle activation using neuromuscular electrical stimulation (NMES) does not improve muscle strength, but increases muscle mass by up-regulating Myhc gene expression. No benefit has been observed on long-term outcomes [155, 158–160]. (3) Functional physical therapy, such as in-bed cycling or functional electrical stimulation (FESCE), is currently thought not to improve muscle strength, muscle mass, or physical outcomes in the short- or long-term, however they are safe [155, 161–163]. (4) Few studies have assessed exercise training post-ICU, and current evidence does not support a clear benefit for physical recovery [164]. Walsh et al. conducted the first RCT assessing combined physical and nutritional rehabilitation during the post-ICU hospital ward, but it showed no improvement in physical recovery outcomes [165] (Table 2).

To explain these results, we could put forward some hypotheses: (a) In a recent negative study evaluating FESCE in critically ill patients receiving mechanical ventilation, RNA sequencing of electrically stimulated *versus* non-stimulated muscle tissue revealed that the intervention's inefficacy was likely due to persistent inflammatory and metabolic dysregulation [161, 166]; (b) Since physical fitness improves physiologically with exercise training over several weeks, it is possible that the dose and/or duration of the interventions were not sufficient to observe long-term results; (c) the exercise training response is highly heterogeneous among individuals (e.g. in elderly, diabetic, or athletic populations), with some subjects experiencing greater improvements than others [167, 168]. Further research should specify the optimal dose (duration, intensity, frequency, and volume) of early mobilization and the role of evoked muscle activation strategies (e.g. NMES) in the physical rehabilitation arsenal. Additionally, studies should investigate the effectiveness of a structured training program, *i.e.* intervention in which patients are engaged in planned (over at least 12 weeks), time-adaptive, individualized, supervised exercise programs [169]. Increasing the cumulative dose of exercise from the ICU stay through post-ICU convalescence may contribute to improved long-term physical outcomes. However, the optimal type of exercise (e.g., aerobic, resistance, balance) and its appropriate dose remains to be determined (Table 2).

#### Nutritional approaches

Following ICU discharge, nutritional intake is generally considered to be insufficient, especially for those who had their enteral nutrition stopped prematurely in the ICU ward and depended thereafter solely on an oral diet [170]. Nevertheless, reaching the protein calorie target, based on formula-based calculations by dieticians, was shown not to be associated with improvement in recovery [171]. Protocol-guided and individualised nutritional support by specialist dietitians has proven its effectiveness among non-critically ill medical patients by lowering mortality [172]. Such a strategy could be applied to survivors after ICU discharge. In addition, as swallowing disorders are common post-ICU, early detection is essential to allow timely adaptation of dietary texture and initiation of compensatory strategies (e.g., supraglottic swallowing). Pharyngeal electrical stimulation via gastric feeding tube shows promise, but requires further validation [45, 46] (Table 2).

## Conclusion

After an initial improvement in the first months after critical illness, muscle weakness persists for years and impacts the long-term prognosis of survivors, increasing healthcare facilities and socio-economic costs. Pre-ICU health status, ICU-related conditions and treatments, and post-ICU management all influence the physical recovery. The pathophysiology of post-ICU muscle weakness is dynamic over time, from the ICU stay through post-ICU convalescence, with some mechanisms fading (e.g. anabolic resistance) while others arise (e.g. impaired regeneration) or persist (e.g. mitochondrial dysfunction). It involves complex dysfunctions spanning the CNS to skeletal muscle, and implies multifaced and interconnected cellular, molecular, and systemic processes. However, these mechanisms remain poorly understood, warranting further investigation in humans through advanced biotechnological tools to uncover novel biological therapeutic strategies. Physical rehabilitation within ICU improves short-term outcomes but its long-term benefits appear limited, potentially due to insufficient cumulative dose, persistent inflammatory and metabolic disturbances in skeletal muscle, and inter-individual variability in exercise response. To date, nutrition- and rehabilitation-based strategies following ICU discharge have not demonstrated evidence of a benefit, but this remains an emerging field and evidence from other applications indicates it warrants further investigation [164]. Interventions targeting post-ICU muscle weakness should commence in the early convalescence phase, before the onset of fibrosis. Multi-approach treatments combining nutritional, physical, and biological approaches during and after the ICU stay should be considered to improve long-term physical outcomes.

## Supplementary Information

Additional file1 (DOCX 14 KB)

Additional file2 (XLSX 13 KB)

## Data Availability

Not applicable

## Abbreviations

ARDS: Acute respiratory distress syndrome
CCI: Chronic critical Illness
CNS: Central nervous system
CLP: Cecal ligation puncture
DAMPs: Damage-associated molecular patterns
EMG: Electromyography
ICU: Intensive care unit
ICUAW: ICU-acquired weakness
IGFBP3: Insulin-like growth factor binding protein 3
ME/CFS: Myalgic encephalomyelitis/chronic fatigue syndrome
miRNA: Micro-RNA
MRC: Medical research council
MSCs: Mesenchymal stem cells
MuSCs: Muscle satellite cells
NMBA: Neuromuscular blocking agents
NMJ: Neuromuscular junction
PICS: Post-intensive care syndrome
PICU: Pediatric ICU
PNS: Peripheral nervous system
QOL: Quality of life
RCT: Randomized controlled trial
rT3: Reverse triiodothyronine
SASP: Senescence-associated secretory phenotype
UPS: Ubiquitin-proteasome system
VO2: Systemic oxygen consumption

## References

[1] Voiriot G, Oualha M, Pierre A, Salmon-Gandonnière C, Gaudet A, Jouan Y, et al. Chronic critical illness and post-intensive care syndrome: from pathophysiology to clinical challenges. Ann Intensive Care. 2022;12(1):58. 35779142 10.1186/s13613-022-01038-0 PMC9250584

[2] Poulsen JB, Rose MH, Jensen BR, Møller K, Perner A. Biomechanical and nonfunctional assessment of physical capacity in male ICU survivors*. Crit Care Med. 2013;41(1):93. 23222267 10.1097/CCM.0b013e31826a3f9e

[3] Van Aerde N, Meersseman P, Debaveye Y, Wilmer A, Gunst J, Casaer MP, et al. Five-year impact of ICU-acquired neuromuscular complications: a prospective, observational study. Intensive Care Med. 2020;46(6):1184–93. 31970446 10.1007/s00134-020-05927-5

[4] Van Aerde N, Meersseman P, Debaveye Y, Wilmer A, Casaer MP, Gunst J, et al. Aerobic exercise capacity in long-term survivors of critical illness: secondary analysis of the post-EPaNIC follow-up study. Intensive Care Med. 2021 10.1007/s00134-021-06541-9 PMC8575347 34750648

[5] Jouan Y, Grammatico-Guillon L, Teixera N, Hassen-Khodja C, Gaborit C, Salmon-Gandonnière C, et al. Healthcare trajectories before and after critical illness: population-based insight on diverse patients clusters. Ann Intensive Care. 2019;9(1):126. 31707487 10.1186/s13613-019-0599-3 PMC6842359

[6] Vanhorebeek I, Latronico N, Van den Berghe G. ICU-acquired weakness. Intensive Care Med. 2020;46(4):637–53. 32076765 10.1007/s00134-020-05944-4 PMC7224132

[7] Fan E, Dowdy DW, Colantuoni E, Mendez-Tellez PA, Sevransky JE, Shanholtz C, et al. Physical complications in acute lung injury survivors: a two-year longitudinal prospective study. Crit Care Med. 2014;42(4):849–59. 24247473 10.1097/CCM.0000000000000040 PMC3959239

[8] Fleischmann-Struzek C, Born S, Kesselmeier M, Ely EW, Töpfer K, Romeike H, et al. Functional dependence following intensive care unit-treated sepsis: three-year follow-up results from the prospective Mid-German sepsis cohort (MSC). Lancet Regional Health Eur. 2024;46:101066. 10.1016/j.lanepe.2024.101066 PMC11415812 39308983

[9] Solverson KJ, Grant C, Doig CJ. Assessment and predictors of physical functioning post-hospital discharge in survivors of critical illness. Ann Intensive Care. 2016;6(1):92. 27646108 10.1186/s13613-016-0187-8 PMC5028364

[10] Poulsen JB, Møller K, Kehlet H, Perner A. Long-term physical outcome in patients with septic shock. Acta Anaesthesiol Scand. 2009;53(6):724–30. 19388891 10.1111/j.1399-6576.2009.01921.x

[11] Yende S, Austin S, Rhodes A, Finfer S, Opal S, Thompson T, et al. Long-term quality of life among survivors of severe sepsis: analyses of two international trials. Crit Care Med. 2016;44(8):1461–7. 26992066 10.1097/CCM.0000000000001658 PMC4949079

[12] Dinglas VD, Friedman LA, Colantuoni E, Mendez-Tellez PA, Shanholtz CB, Ciesla ND, et al. Muscle weakness and 5-year survival in acute respiratory distress syndrome survivors. Crit Care Med. 2017;45(3):446–53. 28067712 10.1097/CCM.0000000000002208 PMC5315580

[13] Mankowski RT, Anton SD, Ghita GL, Brumback B, Cox MC, Mohr AM, et al. Older sepsis survivors suffer persistent disability burden and poor long-term survival. J Am Geriatr Soc. 2020;68(9):1962–9. 32294254 10.1111/jgs.16435 PMC7654284

[14] Wieske L, Dettling-Ihnenfeldt DS, Verhamme C, Nollet F, van Schaik IN, Schultz MJ, et al. Impact of ICU-acquired weakness on post-ICU physical functioning: a follow-up study. Crit Care. 2015;27(19):196. 10.1186/s13054-015-0937-2 PMC4427976 25928709

[15] Lulic-Kuryllo T, Benedini M, Cogliati M, Cudicio A, Guarneri B, Gazzina S, et al. Sex-differences in the longitudinal recovery of neuromuscular function in COVID-19 associated acute respiratory distress syndrome survivors. Frontiers in Medicine [Internet]. 2023 [cited 2024 Jun 28]; 10. Available from: https://consensus.app/papers/sexdifferences-recovery-function-covid19-associated-lulickuryllo/90e4a33a3f92516db9357828cf5b356e/10.3389/fmed.2023.1185479 PMC10330713 37435534

[16] Engelhardt LJ, Grunow JJ, Wollersheim T, Carbon NM, Balzer F, Spranger J, et al. Sex-specific aspects of skeletal muscle metabolism in the clinical context of intensive care unit-acquired weakness. J Clin Med. 2022;11(3):846. 35160299 10.3390/jcm11030846 PMC8836746

[17] Sharshar T, Bastuji-Garin S, Polito A, De Jonghe B, Stevens RD, Maxime V, et al. Hormonal status in protracted critical illness and in-hospital mortality. Crit Care. 2011;15(1):R47. 21291516 10.1186/cc10010 PMC3221977

[18] Dieli-Conwright CM, Spektor TM, Rice JC, Sattler FR, Schroeder ET. Influence of hormone replacement therapy on eccentric exercise induced myogenic gene expression in postmenopausal women. J Appl Physiol. 2009;107(5):1381–8. 19696363 10.1152/japplphysiol.00590.2009 PMC2777804

[19] Sakr Y, Alhussami I, Nanchal R, Wunderink RG, Pellis T, Wittebole X, et al. Being overweight is associated with greater survival in ICU patients: results from the intensive care over nations audit. Crit Care Med. 2015;43(12):2623–32. 26427591 10.1097/CCM.0000000000001310

[20] Goossens C, Marques MB, Derde S, Vander Perre S, Dufour T, Thiessen SE, et al. Premorbid obesity, but not nutrition, prevents critical illness-induced muscle wasting and weakness. J Cachexia Sarcopenia Muscle. 2017;8(1):89–101. 27897405 10.1002/jcsm.12131 PMC5326828

[21] Vankrunkelsven W, Derde S, Gunst J, Vander Perre S, Declerck E, Pauwels L, et al. Obesity attenuates inflammation, protein catabolism, dyslipidaemia, and muscle weakness during sepsis, independent of leptin. J Cachexia Sarcopenia Muscle. 2022;13(1):418–33. 34994068 10.1002/jcsm.12904 PMC8818596

[22] Goossens C, Weckx R, Derde S, Dufour T, Vander Perre S, Pauwels L, et al. Adipose tissue protects against sepsis-induced muscle weakness in mice: from lipolysis to ketones. Crit Care. 2019;23(1):236. 31262340 10.1186/s13054-019-2506-6 PMC6600878

[23] Cox MC, Booth M, Ghita G, Wang Z, Gardner A, Hawkins RB, et al. The impact of sarcopenia and acute muscle mass loss on long-term outcomes in critically ill patients with intra-abdominal sepsis. J Cachexia Sarcopenia Muscle. 2021 10.1002/jcsm.12752 PMC8517344 34196134

[24] Bauer J, Morley JE, Schols AMWJ, Ferrucci L, Cruz-Jentoft AJ, Dent E, et al. Sarcopenia: a time for action an SCWD position paper. J Cachexia Sarcopenia Muscle. 2019;10(5):956–61. 31523937 10.1002/jcsm.12483 PMC6818450

[25] Herridge MS, Batt J, Santos CD. ICU-acquired weakness, morbidity, and death. Am J Respir Crit Care Med. 2014;190(4):360–2. 25127302 10.1164/rccm.201407-1263ED

[26] Urbina T, Canoui-Poitrine F, Hua C, Layese R, Alves A, Ouedraogo R, et al. Long-term quality of life in necrotizing soft-tissue infection survivors: a monocentric prospective cohort study. Ann Intensive Care. 2021;2(11):102. 10.1186/s13613-021-00891-9 PMC8253876 34213694

[27] Needham DM, Wozniak AW, Hough CL, Morris PE, Dinglas VD, Jackson JC, et al. Risk factors for physical impairment after acute lung injury in a national, multicenter study. Am J Respir Crit Care Med. 2014;189(10):1214–24. 24716641 10.1164/rccm.201401-0158OC PMC4061900

[28] Loftus T, Moore F, Moldawer L. ICU-Acquired Weakness, Chronic Critical Illness, and the Persistent Inflammation-Immunosuppression and Catabolism Syndrome. Critical Care Med. 2017. Available from: https://consensus.app/papers/icuacquired-weakness-chronic-critical-illness-loftus/bb5c8685d7ca52b796a930fda1033c3e/10.1097/CCM.0000000000002576 PMC5653275 29028707

[29] Gardner AK, Ghita GL, Wang Z, Ozrazgat-Baslanti T, Raymond SL, Mankowski RT, et al. The development of chronic critical illness determines physical function, quality of life, and long-term survival among early survivors of sepsis in surgical ICUs. Crit Care Med. 2019;47(4):566–73. 30664526 10.1097/CCM.0000000000003655 PMC6422682

[30] Fritzen A, Madsen AB, Kleinert M, Treebak JT, Lundsgaard A, Jensen T, et al. Regulation of autophagy in human skeletal muscle: effects of exercise, exercise training and insulin stimulation. J Physiology. 2016;594:745–61. 10.1113/JP271405 PMC5341711 26614120

[31] Fetterplace K, Beach LJ, MacIsaac C, Presneill J, Edbrooke L, Parry SM, et al. Associations between nutritional energy delivery, bioimpedance spectroscopy and functional outcomes in survivors of critical illness. J Hum Nutr Diet. 2019;32(6):702–12. 31034122 10.1111/jhn.12659

[32] Casaer MP, Wilmer A, Hermans G, Wouters PJ, Mesotten D, Van den Berghe G. Role of disease and macronutrient dose in the randomized controlled EPaNIC trial. Am J Respir Crit Care Med. 2013;187(3):247–55. 23204255 10.1164/rccm.201206-0999OC

[33] null null. Energy-Dense versus Routine Enteral Nutrition in the Critically Ill. New England Journal of Medicine. 2018;379(19):1823–34 10.1056/NEJMoa1811687 30346225

[34] Deane AM, Little L, Bellomo R, Chapman MJ, Davies AR, Ferrie S, et al. Outcomes six months after delivering 100% or 70% of enteral calorie requirements during critical illness (TARGET). A randomized controlled trial. Am J Respir Crit Care Med. 2020;201(7):814–22. 31904995 10.1164/rccm.201909-1810OC

[35] Hermans G, Casaer MP, Clerckx B, Güiza F, Vanhullebusch T, Derde S, et al. Effect of tolerating macronutrient deficit on the development of intensive-care unit acquired weakness: a subanalysis of the EPaNIC trial. Lancet Respir Med. 2013;1(8):621–9. 24461665 10.1016/S2213-2600(13)70183-8

[36] Casaer MP, Mesotten D, Hermans G, Wouters PJ, Schetz M, Meyfroidt G, et al. Early versus late parenteral nutrition in critically Ill adults. N Engl J Med. 2011;365(6):506–17. 21714640 10.1056/NEJMoa1102662

[37] Pierre A, Bourel C, Favory R, Brassart B, Wallet F, Daussin FN, et al. Sepsis-like energy deficit is not sufficient to induce early muscle fiber atrophy and mitochondrial dysfunction in a murine sepsis model. Biology. 2023;12(4):529. 37106730 10.3390/biology12040529 PMC10136327

[38] Gunst J, Derese I, Aertgeerts A, Ververs EJ, Wauters A, Van den Berghe G, et al. Insufficient autophagy contributes to mitochondrial dysfunction, organ failure, and adverse outcome in an animal model of critical illness. Crit Care Med. 2013;41(1):182–94. 23222264 10.1097/CCM.0b013e3182676657

[39] Derde S, Hermans G, Derese I, Güiza F, Hedström Y, Wouters PJ, et al. Muscle atrophy and preferential loss of myosin in prolonged critically ill patients. Crit Care Med. 2012;40(1):79–89. 21926599 10.1097/CCM.0b013e31822d7c18

[40] Reignier J, Plantefeve G, Mira JP, Argaud L, Asfar P, Aissaoui N, et al. Low versus standard calorie and protein feeding in ventilated adults with shock: a randomised, controlled, multicentre, open-label, parallel-group trial (NUTRIREA-3). Lancet Respir Med Internet. 2023. Available from: https://www.thelancet.com/journals/lanres/article/PIIS2213-2600(23)00092-9/fulltext 10.1016/S2213-2600(23)00092-9 36958363

[41] Papazian L, Forel JM, Gacouin A, Penot-Ragon C, Perrin G, Loundou A, et al. Neuromuscular blockers in early acute respiratory distress syndrome. N Engl J Med. 2010;363(12):1107–16. 20843245 10.1056/NEJMoa1005372

[42] null null. Early Neuromuscular Blockade in the Acute Respiratory Distress Syndrome. New Engl J Med. 2019;380(21):1997–2008 10.1056/NEJMoa1901686 PMC6741345 31112383

[43] García-Grimaldo A, Rodríguez-Moguel NC, Godínez-Victoria M, Rodríguez-Llamazares S, Ríos-Ayala MA, Cadeza-Aguilar JD, et al. Associations between Intensive care unit acquired weakness with post-extubation dysphagia and other clinical outcomes-a cohort study in critically ill respiratory patients. Clin Nutr ESPEN. 2025;66:194–201. 39864521 10.1016/j.clnesp.2025.01.044

[44] Schefold JC, Berger D, Zürcher P, Lensch M, Perren A, Jakob SM, et al. Dysphagia in mechanically ventilated ICU patients (DYnAMICS): a prospective observational trial. Crit Care Med. 2017;45(12):2061–9. 29023260 10.1097/CCM.0000000000002765

[45] Likar R, Aroyo I, Bangert K, Degen B, Dziewas R, Galvan O, et al. Management of swallowing disorders in ICU patients - a multinational expert opinion. J Crit Care. 2024;79:154447. 37924574 10.1016/j.jcrc.2023.154447

[46] Zuercher P, Moret CS, Dziewas R, Schefold JC. Dysphagia in the intensive care unit: epidemiology, mechanisms, and clinical management. Crit Care. 2019;23(1):103. 30922363 10.1186/s13054-019-2400-2 PMC6438038

[47] Tanaka K, Watanabe K, Kashiwagi H. Association between postextubation dysphagia and physical function in survivors of critical illness: a retrospective study. Clin Nutr ESPEN. 2022;47:147–51. 35063194 10.1016/j.clnesp.2021.12.031

[48] Devine H, Quasim T, McPeake J, Shaw M, Mccallum L, Mactavish P. Chronic pain in intensive care unit survivors: incidence, characteristics and side-effects up to one-year post-discharge. J Rehabil Med. 2019;51(6):451–5. 31032523 10.2340/16501977-2558

[49] Clavet H, Hébert PC, Fergusson D, Doucette S, Trudel G. Joint contracture following prolonged stay in the intensive care unit. CMAJ. 2008;178(6):691–7. 18332384 10.1503/cmaj.071056 PMC2263098

[50] Goverman J, Mathews K, Goldstein R, Holavanahalli R, Kowalske K, Esselman P, et al. Adult contractures in burn injury: a burn model system national database study. J Burn Care Res. 2017;38(1):e328–36. 27380122 10.1097/BCR.0000000000000380 PMC10032147

[51] Arshad A, Ayaz A, Rehman S, Ukrani RD, Akbar I, Jamil B. Progression of acute kidney injury to chronic kidney disease in sepsis survivors: 1-year follow-up study. J Intensive Care Med. 2021;36(11):1366–70. 32878537 10.1177/0885066620956621

[52] Chaïbi K, Ehooman F, Pons B, Martin-Lefevre L, Boulet E, Boyer A, et al. Long-term outcomes after severe acute kidney injury in critically ill patients: the SALTO study. Ann Intensive Care. 2023;13(1):18. 36907976 10.1186/s13613-023-01108-x PMC10008759

[53] Song IA, Park HY, Oh TK. Sleep disorder and long-term mortality among sepsis survivors: a nationwide cohort study in South Korea. Nat Sci Sleep. 2021;13:979–88. 34234601 10.2147/NSS.S319769 PMC8254539

[54] Pisani MA, Friese RS, Gehlbach BK, Schwab RJ, Weinhouse GL, Jones SF. Sleep in the intensive care unit. Am J Respir Crit Care Med. 2015;191(7):731–8. 25594808 10.1164/rccm.201411-2099CI PMC5447310

[55] Iwashyna TJ, Ely EW, Smith DM, Langa KM. Long-term cognitive impairment and functional disability among survivors of severe sepsis. JAMA. 2010;304(16):1787–94. 20978258 10.1001/jama.2010.1553 PMC3345288

[56] Pandharipande PP, Girard TD, Jackson JC, Morandi A, Thompson JL, Pun BT, et al. Long-term cognitive impairment after critical illness. N Engl J Med. 2013;369(14):1306–16. 24088092 10.1056/NEJMoa1301372 PMC3922401

[57] Parker AM, Sricharoenchai T, Raparla S, Schneck KW, Bienvenu OJ, Needham DM. Posttraumatic stress disorder in critical illness survivors: a metaanalysis. Crit Care Med. 2015;43(5):1121–9. 25654178 10.1097/CCM.0000000000000882

[58] Jackson JC, Pandharipande PP, Girard TD, Brummel NE, Thompson JL, Hughes CG, et al. Depression, post-traumatic stress disorder, and functional disability in survivors of critical illness in the BRAIN-ICU study: a longitudinal cohort study. Lancet Respir Med. 2014;2(5):369–79. 24815803 10.1016/S2213-2600(14)70051-7 PMC4107313

[59] Kamdar BB, Sepulveda KA, Chong A, Lord RK, Dinglas VD, Mendez-Tellez PA, et al. Return to work and lost earnings after acute respiratory distress syndrome: a 5-year prospective, longitudinal study of long-term survivors. Thorax. 2018;73(2):125–33. 28918401 10.1136/thoraxjnl-2017-210217 PMC6002952

[60] Skei NV, Moe K, Nilsen TIL, Aasdahl L, Prescott HC, Damås JK, et al. Return to work after hospitalization for sepsis: a nationwide, registry-based cohort study. Crit Care. 2023;27(1):443. 37968648 10.1186/s13054-023-04737-7 PMC10652599

[61] Haines KJ, Denehy L, Skinner EH, Warrillow S, Berney S. Psychosocial outcomes in informal caregivers of the critically ill: a systematic review. Crit Care Med. 2015;43(5):1112–20. 25654174 10.1097/CCM.0000000000000865

[62] Cameron JI, Chu LM, Matte A, Tomlinson G, Chan L, Thomas C, et al. One-year outcomes in caregivers of critically Ill patients. N Engl J Med. 2016;374(19):1831–41. 27168433 10.1056/NEJMoa1511160

[63] Van den Berghe G. On the neuroendocrinopathy of critical illness. Perspectives for feeding and novel treatments. Am J Respir Crit Care Med. 2016;194(11):1337–48. 27611700 10.1164/rccm.201607-1516CI

[64] Peeters B, Meersseman P, Vander Perre S, Wouters PJ, Debaveye Y, Langouche L, et al. ACTH and cortisol responses to CRH in acute, subacute, and prolonged critical illness: a randomized, double-blind, placebo-controlled, crossover cohort study. Intensive Care Med. 2018;44(12):2048–58. 30374692 10.1007/s00134-018-5427-y PMC6280831

[65] Sav A, Rotondo F, Syro LV, Serna CA, Kovacs K. Pituitary pathology in traumatic brain injury: a review. Pituitary. 2019;22(3):201–11. 30927184 10.1007/s11102-019-00958-8

[66] Vanhorebeek I, Derese I, Gunst J, Wouters PJ, Hermans G, Van den Berghe G. Persisting neuroendocrine abnormalities and their association with physical impairment 5 years after critical illness. Crit Care. 2021;25(1):430. 34915907 10.1186/s13054-021-03858-1 PMC8675467

[67] Wischmeyer PE, Suman OE, Kozar R, Wolf SE, Molinger J, Pastva AM. Role of anabolic testosterone agents and structured exercise to promote recovery in ICU survivors. Curr Opin Crit Care. 2020;26(5):508–15. 32773614 10.1097/MCC.0000000000000757 PMC8367823

[68] Ferrando AA, Sheffield-Moore M, Wolf SE, Herndon DN, Wolfe RR. Testosterone administration in severe burns ameliorates muscle catabolism. Crit Care Med. 2001;29(10):1936–42. 11588456 10.1097/00003246-200110000-00015

[69] Wang J, Wu T. Testosterone improves muscle function of the extensor digitorum longus in rats with sepsis. Biosci Rep. 2020;40(2):BSR20193342. 31967292 10.1042/BSR20193342 PMC7000367

[70] Lincoff AM, Bhasin S, Flevaris P, Mitchell LM, Basaria S, Boden WE, et al. Cardiovascular safety of testosterone-replacement therapy. N Engl J Med. 2023;389(2):107–17. 37326322 10.1056/NEJMoa2215025

[71] Greising SM, Baltgalvis KA, Kosir AM, Moran AL, Warren GL, Lowe DA. Estradiol’s beneficial effect on murine muscle function is independent of muscle activity. J Appl Physiol (1985). 2011;110(1):109–15. 20966194 10.1152/japplphysiol.00852.2010 PMC3253000

[72] Kitajima Y, Ono Y. Estrogens maintain skeletal muscle and satellite cell functions. J Endocrinol. 2016;229(3):267–75. 27048232 10.1530/JOE-15-0476

[73] Enns DL, Tiidus PM. The influence of estrogen on skeletal muscle: sex matters. Sports Med. 2010;40(1):41–58. 20020786 10.2165/11319760-000000000-00000

[74] Mendelson AA, Erickson D, Villar R. The role of the microcirculation and integrative cardiovascular physiology in the pathogenesis of ICU-acquired weakness. Front Physiol. 2023. 10.3389/fphys.2023.1170429. 37234410 10.3389/fphys.2023.1170429 PMC10206327

[75] De Backer D, Donadello K, Sakr Y, Ospina-Tascon G, Salgado D, Scolletta S, et al. Microcirculatory alterations in patients with severe sepsis: impact of time of assessment and relationship with outcome. Crit Care Med. 2013;41(3):791–9. 23318492 10.1097/CCM.0b013e3182742e8b

[76] Donati A, Damiani E, Domizi R, Scorcella C, Carsetti A, Tondi S, et al. Near-infrared spectroscopy for assessing tissue oxygenation and microvascular reactivity in critically ill patients: a prospective observational study. Crit Care. 2016;20(1):311. 27716370 10.1186/s13054-016-1500-5 PMC5045573

[77] Cho DS, Schmitt RE, Dasgupta A, Ducharme AM, Doles JD. Single-cell deconstruction of post-sepsis skeletal muscle and adipose tissue microenvironments. J Cachexia Sarcopenia Muscle. 2020;11(5):1351–63. 32643301 10.1002/jcsm.12596 PMC7567136

[78] Goossens C, Weckx R, Derde S, Van Helleputte L, Schneidereit D, Haug M, et al. Impact of prolonged sepsis on neural and muscular components of muscle contractions in a mouse model. J Cachexia Sarcopenia Muscle. 2021 10.1002/jcsm.12668 PMC8061378 33465304

[79] Dos Santos C, Hussain SNA, Mathur S, Picard M, Herridge M, Correa J, et al. Mechanisms of chronic muscle wasting and dysfunction after an intensive care unit stay. A pilot study. Am J Respir Crit Care Med. 2016;194(7):821–30. 27058306 10.1164/rccm.201512-2344OC

[80] Gruet M, Temesi J, Rupp T, Levy P, Millet GY, Verges S. Stimulation of the motor cortex and corticospinal tract to assess human muscle fatigue. Neuroscience. 2013;12(231):384–99. 10.1016/j.neuroscience.2012.10.058 23131709

[81] Morel J, Infantino P, Gergelé L, Lapole T, Souron R, Millet GY. Prevalence of self-reported fatigue in intensive care unit survivors 6 months-5 years after discharge. Sci Rep. 2022;12(1):5631. 35379874 10.1038/s41598-022-09623-w PMC8979153

[82] Hussain N, Samuelsson CM, Drummond A, Persson CU. Prevalence of fatigue at one-year follow-up from the Gothenburg recovery and rehabilitation after COVID-19 and intensive care unit study. Sci Rep. 2022;12(1):11501. 35821226 10.1038/s41598-022-14787-6 PMC9276681

[83] Egger M, Wimmer C, Stummer S, Reitelbach J, Bergmann J, Müller F, et al. Reduced health-related quality of life, fatigue, anxiety and depression affect COVID-19 patients in the long-term after chronic critical illness. Sci Rep. 2024;14(1):3016. 38321074 10.1038/s41598-024-52908-5 PMC10847136

[84] Mayer KP, Ismaeel A, Kalema AG, Montgomery-Yates AA, Soper MK, Kern PA, et al. Persistent fatigue, weakness, and aberrant muscle mitochondria in survivors of critical COVID-19. Crit Care Explor. 2024;6(10):e1164. 39412208 10.1097/CCE.0000000000001164 PMC11487221

[85] Wan J, Qin Z, Wang PY, Sun Y, Liu X. Muscle fatigue: general understanding and treatment. Exp Mol Med. 2017;49(10):e384–e384. 28983090 10.1038/emm.2017.194 PMC5668469

[86] Walitt B, Singh K, LaMunion SR, Hallett M, Jacobson S, Chen K, et al. Deep phenotyping of post-infectious myalgic encephalomyelitis/chronic fatigue syndrome. Nat Commun. 2024;15(1):907. 38383456 10.1038/s41467-024-45107-3 PMC10881493

[87] Souron R, Morel J, Gergelé L, Infantino P, Brownstein CG, Lapole T, et al. Relationship between intensive care unit-acquired weakness, fatigability and fatigue: What role for the central nervous system? J Crit Care. 2021;62:101–10. 33316555 10.1016/j.jcrc.2020.11.019

[88] Bierbrauer J, Koch S, Olbricht C, Hamati J, Lodka D, Schneider J, et al. Early type II fiber atrophy in intensive care unit patients with nonexcitable muscle membrane. Crit Care Med. 2012;40(2):647–50. 21963579 10.1097/CCM.0b013e31823295e6

[89] Wollersheim T, Woehlecke J, Krebs M, Hamati J, Lodka D, Luther-Schroeder A, et al. Dynamics of myosin degradation in intensive care unit-acquired weakness during severe critical illness. Intensive Care Med. 2014;40(4):528–38. 24531339 10.1007/s00134-014-3224-9

[90] Owen AM, Patel SP, Smith JD, Balasuriya BK, Mori SF, Hawk GS, et al. Chronic muscle weakness and mitochondrial dysfunction in the absence of sustained atrophy in a preclinical sepsis model. Elife. 2019;03:8. 10.7554/eLife.49920 PMC6890461 31793435

[91] Crowell KT, Lang CH. Contractility and myofibrillar content in skeletal muscle are decreased during post-sepsis recovery, but not during the acute phase of sepsis. Shock. 2021;55(5):649–59. 32433214 10.1097/SHK.0000000000001555

[92] Zauner A, Nimmerrichter P, Anderwald C, Bischof M, Schiefermeier M, Ratheiser K, et al. Severity of insulin resistance in critically ill medical patients. Metabolism. 2007;56(1):1–5. 17161218 10.1016/j.metabol.2006.08.014

[93] Weber-Carstens S, Schneider J, Wollersheim T, Assmann A, Bierbrauer J, Marg A, et al. Critical illness myopathy and GLUT4: significance of insulin and muscle contraction. Am J Respir Crit Care Med. 2013;187(4):387–96. 23239154 10.1164/rccm.201209-1649OC

[94] Gornik I, Vujaklija-Brajković A, Renar IP, Gašparović V. A prospective observational study of the relationship of critical illness associated hyperglycaemia in medical ICU patients and subsequent development of type 2 diabetes. Crit Care. 2010;14(4):R130. 20615210 10.1186/cc9101 PMC2945097

[95] Cree MG, Fram RY, Barr D, Chinkes D, Wolfe RR, Herndon DN. Insulin resistance, secretion and breakdown are increased 9 months following severe burn injury. Burns. 2009;35(1):63–9. 18672331 10.1016/j.burns.2008.04.010 PMC3503248

[96] Gauglitz GG, Herndon DN, Kulp GA, Meyer WJ, Jeschke MG. Abnormal insulin sensitivity persists up to three years in pediatric patients post-burn. J Clin Endocrinol Metab. 2009;94(5):1656–64. 19240154 10.1210/jc.2008-1947 PMC2684478

[97] Carbon NM, Engelhardt LJ, Wollersheim T, Grunow JJ, Spies CD, Märdian S, et al. Impact of protocol-based physiotherapy on insulin sensitivity and peripheral glucose metabolism in critically ill patients. J Cachexia Sarcopenia Muscle. 2022;13(2):1045–53. 35075782 10.1002/jcsm.12920 PMC8978012

[98] Kulkarni AS, Gubbi S, Barzilai N. Benefits of metformin in attenuating the hallmarks of aging. Cell Metab. 2020;32(1):15–30. 32333835 10.1016/j.cmet.2020.04.001 PMC7347426

[99] Gasparetto A, Corbucci GG, Candiani A, Gohil K, Edwards RHT. Effect of tissue hypoxia and septic shock on human skeletal muscle mitochondria. Lancet. 1983;322(8365–8366):1486. 10.1016/s0140-6736(83)90823-1 6140567

[100] Preau S, Vodovar D, Jung B, Lancel S, Zafrani L, Flatres A, et al. Energetic dysfunction in sepsis: a narrative review. Ann Intensive Care. 2021;11(1):104. 34216304 10.1186/s13613-021-00893-7 PMC8254847

[101] Brealey D, Brand M, Hargreaves I, Heales S, Land J, Smolenski R, et al. Association between mitochondrial dysfunction and severity and outcome of septic shock. Lancet. 2002;360(9328):219–23. 12133657 10.1016/S0140-6736(02)09459-X

[102] Klawitter F, Ehler J, Bajorat R, Patejdl R. Mitochondrial dysfunction in intensive care unit-acquired weakness and critical illness myopathy: a narrative review. Int J Mol Sci. 2023;24(6):5516. 36982590 10.3390/ijms24065516 PMC10052131

[103] Walsh CJ, Batt J, Herridge MS, Mathur S, Bader GD, Hu P, et al. Transcriptomic analysis reveals abnormal muscle repair and remodeling in survivors of critical illness with sustained weakness. Sci Rep. 2016;14(6):29334. 10.1038/srep29334 PMC4944143 27411715

[104] Walsh CJ, Escudero King C, Gupta M, Plant PJ, Herridge MJ, Mathur S, et al. MicroRNA regulatory networks associated with abnormal muscle repair in survivors of critical illness. J Cachexia Sarcopenia Muscle. 2022;13(2):1262–76. 35092190 10.1002/jcsm.12903 PMC8977950

[105] Mart MF, Ely EW, Tolle JJ, Patel MB, Brummel NE. Physiologic responses to exercise in survivors of critical illness: an exploratory pilot study. Intensive Care Med Exp. 2022;10(1):35. 36008625 10.1186/s40635-022-00461-8 PMC9410741

[106] Monzel AS, Enríquez JA, Picard M. Multifaceted mitochondria: moving mitochondrial science beyond function and dysfunction. Nat Metab. 2023;5(4):546–62. 37100996 10.1038/s42255-023-00783-1 PMC10427836

[107] Chen X, Chen M, Yang Y, Xu C, Lu H, Xu Y, et al. Lipopolysaccharide-preconditioned mesenchymal stem cell transplantation attenuates critical persistent inflammation immune suppression and catabolism syndrome in mice. Shock. 2022;58(5):417–25. 36155397 10.1097/SHK.0000000000001993

[108] Martín-Vicente P, López-Martínez C, Rioseras B, Albaiceta GM. Activation of senescence in critically ill patients: mechanisms, consequences and therapeutic opportunities. Ann Intensiv Care. 2024;14(1):2. 10.1186/s13613-023-01236-4 PMC10769968 38180573

[109] Merdji H, Kassem M, Chomel L, Clere-Jehl R, Helms J, Kurihara K, et al. Septic shock as a trigger of arterial stress-induced premature senescence: a new pathway involved in the post sepsis long-term cardiovascular complications. Vascul Pharmacol. 2021;27:106922. 10.1016/j.vph.2021.106922 34592427

[110] Daussin FN, Boulanger E, Lancel S. From mitochondria to sarcopenia: Role of inflammaging and RAGE-ligand axis implication. Exp Gerontol. 2021;20:111247. 10.1016/j.exger.2021.111247 33484891

[111] Justice JN, Nambiar AM, Tchkonia T, LeBrasseur NK, Pascual R, Hashmi SK, et al. Senolytics in idiopathic pulmonary fibrosis: results from a first-in-human, open-label, pilot study. EBioMedicine. 2019;40:554–63. 30616998 10.1016/j.ebiom.2018.12.052 PMC6412088

[112] Coppens G, Vanhorebeek I, Verlinden I, Derese I, Wouters PJ, Joosten KF, et al. Assessment of aberrant DNA methylation two years after paediatric critical illness: a pre-planned secondary analysis of the international PEPaNIC trial. Epigenetics. 2023;18(1):2146966. 36384393 10.1080/15592294.2022.2146966 PMC9980627

[113] Van Dyck L, Güiza F, Derese I, Pauwels L, Casaer MP, Hermans G, et al. DNA methylation alterations in muscle of critically ill patients. J Cachexia Sarcopenia Muscle. 2022;13(3):1731–40. 35274472 10.1002/jcsm.12970 PMC9178166

[114] Takala J, Ruokonen E, Webster NR, Nielsen MS, Zandstra DF, Vundelinckx G, et al. Increased mortality associated with growth hormone treatment in critically Ill adults. N Engl J Med. 1999;341(11):785–92. 10477776 10.1056/NEJM199909093411102

[115] Chapple LAS, Kouw IWK, Summers MJ, Weinel LM, Gluck S, Raith E, et al. Muscle protein synthesis after protein administration in critical illness. Am J Respir Crit Care Med. 2022;206(6):740–9. 35584344 10.1164/rccm.202112-2780OC

[116] Gamrin-Gripenberg L, Sundström-Rehal M, Olsson D, Grip J, Wernerman J, Rooyackers O. An attenuated rate of leg muscle protein depletion and leg free amino acid efflux over time is seen in ICU long-stayers. Crit Care. 2018;22(1):13. 29361961 10.1186/s13054-017-1932-6 PMC5782367

[117] Crowell KT, Soybel DI, Lang CH. Restorative mechanisms regulating protein balance in skeletal muscle during recovery from sepsis. Shock. 2017;47(4):463–73. 27749759 10.1097/SHK.0000000000000762 PMC5348274

[118] Klaude M, Fredriksson K, Tjäder I, Hammarqvist F, Ahlman B, Rooyackers O, et al. Proteasome proteolytic activity in skeletal muscle is increased in patients with sepsis. Clin Sci (Lond). 2007;112(9):499–506. 17117920 10.1042/CS20060265

[119] Preau S, Ambler M, Sigurta A, Kleyman A, Dyson A, Hill NE, et al. Protein recycling and limb muscle recovery after critical illness in slow- and fast-twitch limb muscle. Am J Physiol Regul Integr Comp Physiol. 2019;316(5):R584–93. 30789789 10.1152/ajpregu.00221.2018

[120] Vana PG, LaPorte HM, Wong YM, Kennedy RH, Gamelli RL, Majetschak M. Proteasome inhibition after burn injury. J Burn Care Res. 2016;37(4):207–15. 26204383 10.1097/BCR.0000000000000280 PMC4721944

[121] Kitajima Y, Yoshioka K, Suzuki N. The ubiquitin-proteasome system in regulation of the skeletal muscle homeostasis and atrophy: from basic science to disorders. J Physiol Sci. 2020;70(1):40. 32938372 10.1186/s12576-020-00768-9 PMC10717345

[122] Masiero E, Agatea L, Mammucari C, Blaauw B, Loro E, Komatsu M, et al. Autophagy is required to maintain muscle mass. Cell Metab. 2009;10(6):507–15. 19945408 10.1016/j.cmet.2009.10.008

[123] Yin X, Xin H, Mao S, Wu G, Guo L. The role of autophagy in sepsis: protection and injury to organs. Front Physiol. 2019;23(10):1071. 10.3389/fphys.2019.01071 PMC6716215 31507440

[124] Rivera JC, Abrigo J, Tacchi F, Simon F, Brandan E, Santos RA, et al. Angiotensin-(1–7) prevents lipopolysaccharide-induced autophagy via the mas receptor in skeletal muscle. Int J Mol Sci. 2020;21(24):9344. 33302427 10.3390/ijms21249344 PMC7762589

[125] Yin D, Lin D, Xie Y, Gong A, Jiang P, Wu J. Neuregulin-1β alleviates sepsis-induced skeletal muscle atrophy by inhibiting autophagy via AKT/mTOR signaling pathway in rats. Shock. 2022;57(3):397–407. 34559744 10.1097/SHK.0000000000001860

[126] Vanhorebeek I, Gunst J, Derde S, Derese I, Boussemaere M, Güiza F, et al. Insufficient activation of autophagy allows cellular damage to accumulate in critically Ill patients. J Clin Endocrinol Metab. 2011;96(4):E633–45. 21270330 10.1210/jc.2010-2563

[127] Takahashi W, Watanabe E, Fujimura L, Watanabe-Takano H, Yoshidome H, Swanson PE, et al. Kinetics and protective role of autophagy in a mouse cecal ligation and puncture-induced sepsis. Crit Care. 2013;17(4):R160. 23883625 10.1186/cc12839 PMC4056358

[128] Chen J, Min S, Xie F, Yang J, Wang X. Enhancing autophagy protects against sepsis-induced neuromuscular dysfunction associated with qualitative changes to acetylcholine receptors. Shock. 2019;52(1):111–21. 30286033 10.1097/SHK.0000000000001189

[129] Leduc-Gaudet JP, Miguez K, Cefis M, Faitg J, Moamer A, Chaffer TJ, et al. Autophagy ablation in skeletal muscles worsens sepsis-induced muscle wasting, impairs whole-body metabolism, and decreases survival. iScience. 2023;26(8):107475. 37588163 10.1016/j.isci.2023.107475 PMC10425945

[130] Griffith DM, Lewis S, Rossi AG, Rennie J, Salisbury L, Merriweather JL, et al. Systemic inflammation after critical illness: relationship with physical recovery and exploration of potential mechanisms. Thorax. 2016;71(9):820–9. 27118812 10.1136/thoraxjnl-2015-208114

[131] Laitano O, Robinson GP, Garcia CK, Mattingly AJ, Sheikh LH, Murray KO, et al. Skeletal muscle interleukin-6 contributes to the innate immune response in septic mice. Shock. 2021;55(5):676–85. 32826815 10.1097/SHK.0000000000001641 PMC8607997

[132] Rehmann R, Enax-Krumova E, Meyer-Frießem CH, Schlaffke L. Quantitative muscle MRI displays clinically relevant myostructural abnormalities in long-term ICU-survivors: a case-control study. BMC Med Imaging. 2023;23(1):38. 36934222 10.1186/s12880-023-00995-7 PMC10024415

[133] Nakanishi N, Ono Y, Miyazaki Y, Moriyama N, Fujioka K, Yamashita K, et al. Sepsis causes neutrophil infiltration in muscle leading to muscle atrophy and weakness in mice. Front Immunol. 2022;13:950646. 36389802 10.3389/fimmu.2022.950646 PMC9659852

[134] Farup J, Madaro L, Puri PL, Mikkelsen UR. Interactions between muscle stem cells, mesenchymal-derived cells and immune cells in muscle homeostasis, regeneration and disease. Cell Death Dis. 2015;6(7):e1830. 26203859 10.1038/cddis.2015.198 PMC4650743

[135] Tao Y, Song L, Xiao H, Liu C. Inference and analysis of cell-cell communication of post-sepsis skeletal muscle based on single-cell RNA-seq. Human Gene. 2023;1(38):201236.

[136] Pierre A, Lancel S, Preau S. Organ crosstalk and dysfunction in Sepsis: harnessing emerging biotechnologies for future breakthroughs. Ann Intensive Care. 2024;14(1):161. 39438362 10.1186/s13613-024-01398-9 PMC11496395

[137] Mann CJ, Perdiguero E, Kharraz Y, Aguilar S, Pessina P, Serrano AL, et al. Aberrant repair and fibrosis development in skeletal muscle. Skelet Muscle. 2011;4(1):21. 10.1186/2044-5040-1-21 PMC3156644 21798099

[138] Rocheteau P, Chatre L, Briand D, Mebarki M, Jouvion G, Bardon J, et al. Sepsis induces long-term metabolic and mitochondrial muscle stem cell dysfunction amenable by mesenchymal stem cell therapy. Nat Commun. 2015;15(6):10145. 10.1038/ncomms10145 PMC4682118 26666572

[139] Schmitt RE, Dasgupta A, Arneson-Wissink PC, Datta S, Ducharme AM, Doles JD. Muscle stem cells contribute to long-term tissue repletion following surgical sepsis. J Cachexia, Sarcopenia Muscle. 2023. 10.1002/jcsm.13214. 36883680 10.1002/jcsm.13214 PMC10235871

[140] Takegaki J, Sase K, Kono Y, Nakano D, Fujita T, Konishi S, et al. Intramuscular injection of mesenchymal stem cells activates anabolic and catabolic systems in mouse skeletal muscle. Sci Rep. 2021;11(1):21224. 34707171 10.1038/s41598-021-00627-6 PMC8551189

[141] Cruz-Jentoft AJ, Bahat G, Bauer J, Boirie Y, Bruyère O, Cederholm T, et al. Sarcopenia: revised European consensus on definition and diagnosis. Age Ageing. 2019;48(4):601. 31081853 10.1093/ageing/afz046 PMC6593317

[142] Deutz NEP, Bauer JM, Barazzoni R, Biolo G, Boirie Y, Bosy-Westphal A, et al. Protein intake and exercise for optimal muscle function with aging: recommendations from the ESPEN expert group. Clin Nutr. 2014;33(6):929–36. 24814383 10.1016/j.clnu.2014.04.007 PMC4208946

[143] Balas MC, Vasilevskis EE, Olsen KM, Schmid KK, Shostrom V, Cohen MZ, et al. Effectiveness and safety of the awakening and breathing coordination, delirium monitoring/management, and early exercise/mobility bundle. Crit Care Med. 2014;42(5):1024–36. 24394627 10.1097/CCM.0000000000000129 PMC4105208

[144] Pun BT, Balas MC, Barnes-Daly MA, Thompson JL, Aldrich JM, Barr J, et al. Caring for critically Ill patients with the ABCDEF bundle: results of the ICU liberation collaborative in over 15,000 adults. Crit Care Med. 2019;47(1):3–14. 30339549 10.1097/CCM.0000000000003482 PMC6298815

[145] Kawakami D, Fujitani S, Koga H, Dote H, Takita M, Takaba A, et al. Evaluation of the impact of ABCDEF bundle compliance rates on postintensive care syndrome: a secondary analysis study*. Crit Care Med. 2023;51:1685–96. 37971720 10.1097/CCM.0000000000005980

[146] Schweickert WD, Pohlman MC, Pohlman AS, Nigos C, Pawlik AJ, Esbrook CL, et al. Early physical and occupational therapy in mechanically ventilated, critically ill patients: a randomised controlled trial. The Lancet. 2009;373(9678):1874–82. 10.1016/S0140-6736(09)60658-9 PMC9906655 19446324

[147] Burtin C, Clerckx B, Robbeets C, Ferdinande P, Langer D, Troosters T, et al. Early exercise in critically ill patients enhances short-term functional recovery. Crit Care Med. 2009;37(9):2499–505. 19623052 10.1097/CCM.0b013e3181a38937

[148] Fuke R, Hifumi T, Kondo Y, Hatakeyama J, Takei T, Yamakawa K, et al. Early rehabilitation to prevent postintensive care syndrome in patients with critical illness: a systematic review and meta-analysis. BMJ Open. 2018;8(5):e019998. 29730622 10.1136/bmjopen-2017-019998 PMC5942437

[149] Wright SE, Thomas K, Watson G, Baker C, Bryant A, Chadwick TJ, et al. Intensive versus standard physical rehabilitation therapy in the critically ill (EPICC): a multicentre, parallel-group, randomised controlled trial. Thorax. 2018;73(3):213–21. 28780504 10.1136/thoraxjnl-2016-209858 PMC5870467

[150] Fossat G, Baudin F, Courtes L, Bobet S, Dupont A, Bretagnol A, et al. Effect of in-bed leg cycling and electrical stimulation of the quadriceps on global muscle strength in critically Ill adults: a randomized clinical trial. JAMA. 2018;320(4):368–78. 30043066 10.1001/jama.2018.9592 PMC6583091

[151] Takaoka A, Utgikar R, Rochwerg B, Cook DJ, Kho ME. The efficacy and safety of in-intensive care unit leg-cycle ergometry in critically Ill adults. A systematic review and meta-analysis. Ann Am Thorac Soc. 2020;17(10):1289–307. 32628501 10.1513/AnnalsATS.202001-059OC

[152] Waldauf P, Jiroutková K, Krajčová A, Puthucheary Z, Duška F. Effects of rehabilitation interventions on clinical outcomes in critically Ill patients: systematic review and meta-analysis of randomized controlled trials. Crit Care Med. 2020;48(7):1055–65. 32345834 10.1097/CCM.0000000000004382

[153] null null. Early Active Mobilization during Mechanical Ventilation in the ICU. New England J Med. 2022 9;387(19):1747–58 10.1056/NEJMoa2209083 36286256

[154] Patel BK, Wolfe KS, Patel SB, Dugan KC, Esbrook CL, Pawlik AJ, et al. Effect of early mobilisation on long-term cognitive impairment in critical illness in the USA: a randomised controlled trial. Lancet Respir Med. (2023). Available from: https://www.thelancet.com/journals/lanres/article/PIIS2213-2600(22)00489-1/fulltext 10.1016/S2213-2600(22)00489-1 PMC10238598 36693400

[155] Jiroutková K, Duška F, Waldauf P. Should new data on rehabilitation interventions in critically Ill patients change clinical practice? Updated meta-analysis of randomized controlled trials. Crit Care Med. 2024 10.1097/CCM.0000000000006259 38501932

[156] Paton M, Chan S, Tipping CJ, Stratton A, Serpa Neto A, Lane R, et al. The effect of mobilization at 6 months after critical illness — meta-analysis. NEJM Evid. 2023;2(2):EVIDoa2200234. 38320036 10.1056/EVIDoa2200234

[157] Renner C, Jeitziner MM, Albert M, Brinkmann S, Diserens K, Dzialowski I, et al. Guideline on multimodal rehabilitation for patients with post-intensive care syndrome. Crit Care. 2023;27(1):301. 37525219 10.1186/s13054-023-04569-5 PMC10392009

[158] Segers J, Vanhorebeek I, Langer D, Charususin N, Wei W, Frickx B, et al. Early neuromuscular electrical stimulation reduces the loss of muscle mass in critically ill patients - a within subject randomized controlled trial. J Crit Care. 2020;28(62):65–71. 10.1016/j.jcrc.2020.11.018 33285371

[159] Eggmann S, Verra ML, Luder G, Takala J, Jakob SM. Effects of early, combined endurance and resistance training in mechanically ventilated, critically ill patients: a study protocol for a randomised controlled trial. Trials. 2016;15(17):403. 10.1186/s13063-016-1533-8 PMC4986184 27527501

[160] Wollersheim T, Grunow JJ, Carbon NM, Haas K, Malleike J, Ramme SF, et al. Muscle wasting and function after muscle activation and early protocol-based physiotherapy: an explorative trial. J Cachexia Sarcopenia Muscle. 2019;10(4):734–47. 31016887 10.1002/jcsm.12428 PMC6711421

[161] Waldauf P, Hrušková N, Blahutova B, Gojda J, Urban T, Krajčová A, et al. Functional electrical stimulation-assisted cycle ergometry-based progressive mobility programme for mechanically ventilated patients: randomised controlled trial with 6 months follow-up. Thorax. 2021;76(7):664–71. 33931570 10.1136/thoraxjnl-2020-215755 PMC8223653

[162] Berney S, Hopkins RO, Rose JW, Koopman R, Puthucheary Z, Pastva A, et al. Functional electrical stimulation in-bed cycle ergometry in mechanically ventilated patients: a multicentre randomised controlled trial. Thorax. 2021;76(7):656–63. 33323480 10.1136/thoraxjnl-2020-215093

[163] Kho ME, Berney S, Pastva AM, Kelly L, Reid JC, Burns KEA, et al. Early in-bed cycle ergometry in mechanically ventilated patients. NEJM Evid. 2024;3(7):EVIDoa2400137. 38865147 10.1056/EVIDoa2400137

[164] Connolly B, Salisbury L, O’Neill B, Geneen L, Douiri A, Grocott MPW, et al. Exercise rehabilitation following intensive care unit discharge for recovery from critical illness: executive summary of a cochrane collaboration systematic review. J Cachexia Sarcopenia Muscle. 2016;7(5):520–6. 27891297 10.1002/jcsm.12146 PMC5114628

[165] Walsh TS, Salisbury LG, Merriweather JL, Boyd JA, Griffith DM, Huby G, et al. Increased hospital-based physical rehabilitation and information provision after intensive care unit discharge: the RECOVER randomized clinical trial. JAMA Intern Med. 2015;175(6):901–10. 25867659 10.1001/jamainternmed.2015.0822

[166] Jameson TSO, Caldow MK, Stephens F, Denehy L, Lynch GS, Koopman R, et al. Inflammation and altered metabolism impede efficacy of functional electrical stimulation in critically ill patients. Crit Care. 2023;27(1):428. 37932834 10.1186/s13054-023-04664-7 PMC10629203

[167] Sparks LM. Exercise training response heterogeneity: physiological and molecular insights. Diabetologia. 2017;60(12):2329–36. 29032385 10.1007/s00125-017-4461-6

[168] Amar D, Gay NR, Jean-Beltran PM, Bae D, Dasari S, Dennis C, et al. Temporal dynamics of the multi-omic response to endurance exercise training. Nature. 2024;629(8010):174–83. 38693412 10.1038/s41586-023-06877-w PMC11062907

[169] Umpierre D, Ribeiro PAB, Kramer CK, Leitão CB, Zucatti ATN, Azevedo MJ, et al. Physical activity advice only or structured exercise training and association with HbA1c levels in type 2 diabetes: a systematic review and meta-analysis. JAMA. 2011;305(17):1790–9. 21540423 10.1001/jama.2011.576

[170] Ridley EJ, Chapple LAS, Chapman MJ. Nutrition intake in the post-ICU hospitalization period. Curr Opin Clin Nutr Metab Care. 2020;23(2):111–5. 31977335 10.1097/MCO.0000000000000637

[171] Slingerland-Boot R, van der Heijden I, Schouten N, Driessen L, Meijer S, Mensink M, et al. Prospective observational cohort study of reached protein and energy targets in general wards during the post-intensive care period: the PROSPECT-I study. Clin Nutr. 2022;41(10):2124–34. 36067584 10.1016/j.clnu.2022.07.031

[172] Schuetz P, Fehr R, Baechli V, Geiser M, Deiss M, Gomes F, et al. Individualised nutritional support in medical inpatients at nutritional risk: a randomised clinical trial. Lancet. 2019;393(10188):2312–21. 31030981 10.1016/S0140-6736(18)32776-4

