# Supplementary material for: Muscle weakness after critical illness: unravelling biological mechanisms and clinical hurdles
Source: Crit Care. 2025 Jun 17;29:248. doi: 10.1186/s13054-025-05462-z (PMC12175454; doi:10.1186/s13054-025-05462-z)
Supplement: Supplementary file 1 — Additional file1 (DOCX 14 KB) [file 13054_2025_5462_MOESM1_ESM.docx]

# Additional file 1

**Methods section**

This review addresses the health impact of post-ICU muscle weakness. It evaluates the influence of clinical factors on long-term physical outcomes in medical and surgical ICU survivors. Then, it summarises the physiological and biological processes underlying this persistent muscle weakness. For each established or putative mechanism, it highlights potential innovative therapeutic approaches and further research directions. Finally, it addresses the issues of nutrition and physical rehabilitation for improving long-term physical outcomes.

To investigate these questions, a narrative literature review was conducted using the PubMed database, covering publications from January 2000 to April 2025. The search strategy was based on iterative combinations of MeSH terms and free-text keywords relevant to muscle weakness following critical illness. Key terms included: *ICU-acquired weakness, post-ICU muscle weakness, post-intensive care syndrome, intensive care unit, critical illness, ICU survivors, sepsis, acute respiratory distress syndrome, insulin resistance, mitochondrial dysfunction, cellular senescence, epigenetics, anabolism, catabolism, autophagy, ubiquitin proteasome system, inflammation, regeneration, nutrition, rehabilitation, early mobilization, neuromuscular electrical stimulation*. Boolean operators (AND, OR) were applied to refine and expand the search. Articles adult medical and surgical ICU human populations and animal models were considered, with a focus on original research.
